# Systematic membrane thickness variation across cellular organelles revealed by cryo-ET

**DOI:** 10.1083/jcb.202504053

**Published:** 2025-11-04

**Authors:** Desislava Glushkova, Stefanie Böhm, Martin Beck

**Affiliations:** 1 Department of Molecular Sociology, Max Planck Institute of Biophysics, Frankfurt am Main, Germany; 2 IMPRS on Cellular Biophysics, Frankfurt am Main, Germany; 3 Institute of Biochemistry, Goethe University Frankfurt, Frankfurt am Main, Germany

## Abstract

In eukaryotes, membrane-bound organelles create distinct molecular environments. The compartmentalizing lipid bilayer is a dynamic composite material whose thickness and curvature modulate the structure and function of membrane proteins. In vitro, bilayer thickness correlates with lipid composition. Cellular membranes in situ, however, are continuously remodeled, and the spatial variation of their biophysical properties remains understudied. Here, we present a computational approach to measure local membrane thickness in cryo-electron tomograms. Our analysis of *Chlamydomonas reinhardtii* and human cells reveals systematic thickness variations within and across organelles. Notably, we observe thickness gradients across the Golgi apparatus that orthogonally support long-standing models of differential sorting of transmembrane proteins based on hydrophobic matching. Our publicly available workflow readily integrates within existing tomogram analysis pipelines and, when applied across experimental systems, provides a quantitative foundation for exploring relationships between membrane thickness and function in native cellular environments.

## Teaser

Glushkova et al. develop a publicly available computational method to measure the thickness of biological membranes in cryo-electron tomograms. Analysis of algae and human cells reveals systematic membrane thickness variations within and across organelles, including thickness gradients across the Golgi apparatus that support long-standing protein sorting models.

## Introduction

Cellular membranes are composite materials, consisting of a lipid bilayer and membrane proteins, which serve as essential sites for biochemical processes in all living organisms (Alberts et al., 2002). The lipid composition of membranes affects their biophysical properties, including curvature (Pinot et al., 2014), thickness (Renne and Ernst, 2023; Sezgin et al., 2017), fluidity (Ernst et al., 2016; Renne and Ernst, 2023), and compressibility (Pinot et al., 2014; Renne and Ernst, 2023). Both direct lipid–protein interactions and indirect changes in membrane properties regulate the function of membrane proteins and ultimately affect diverse biological pathways (Ballweg et al., 2020; Botelho et al., 2006; Bushell et al., 2019; Cybulski et al., 2015; Johannsson et al., 1981; Starling et al., 1993; Wu and Rapoport, 2021).

Eukaryotic cells invest substantial resources in generating a diverse lipidome, comprising more than a thousand distinct species (Harayama and Riezman, 2018), with multiple lipid metabolic pathways underlying the diversity of membrane lipid compositions across species (Hannich et al., 2011; Yamashita et al., 2014), tissues, and cell types (Grösch et al., 2012; Surma et al., 2021; Yamashita et al., 2014). Lipid composition can also change dynamically during various cellular processes: HeLa cells actively regulate both the composition and spatial distribution of lipids during cell division, altering their membrane mechanical properties (Atilla-Gokcumen et al., 2014). Similarly, hormonal tissue differentiation involves modifications to the lipidome of epithelial cells, including changes in the phospholipid and sphingolipid profiles (Postle et al., 2006; Sampaio et al., 2011).

Beyond cell type differences, organelle membranes within individual cells possess distinctive lipid “fingerprints.” Lipidomic analyses of subcellular fractions have revealed nonhomogeneous distributions of phospholipids and sterols across eukaryotic cellular compartments (Sarmento et al., 2023; van Meer et al., 2008), with some lipids primarily found in specific organelles, e.g., cardiolipin in the inner mitochondrial membrane (Gaspard and McMaster, 2015) and lysobisphosphatidic acid in late endosomes (Chevallier et al., 2008). Although the endoplasmic reticulum serves as the primary site for phospholipid and sterol synthesis, its own membranes contain relatively low concentrations of the latter, as cholesterol is rapidly shuttled through the secretory pathway to the Golgi, where sphingolipids are synthesized. Both sphingolipids and cholesterol then get transported to the plasma membrane (PM), where they contribute to creating a tightly packed, relatively impermeable barrier around the cell (Harayama and Riezman, 2018; Sarmento et al., 2023).

Despite significant advances in understanding lipid distribution across cell types and cellular compartments, lipidomic approaches face several limitations: cell lysis eliminates spatial information on membrane organization; obtaining pure organelle fractions can be challenging, particularly for membranes of similar size and density; and the analytical outcomes are sensitive to variations in lipid extraction protocols (Sarmento et al., 2023). Furthermore, the more elaborate morphology of many organelles, e.g., the Golgi cisternae, is lost during isolation.

Organelle-specific lipid compositions not only define membrane identity but also create distinct physicochemical environments that influence protein function and intracellular trafficking. The Golgi apparatus serves as a compelling example: Munro and colleagues have proposed that variations in membrane thickness drive the partitioning of transmembrane proteins into distinct Golgi sub-compartments (Bretscher and Munro, 1993; Munro, 1998), thus minimizing hydrophobic mismatch and its associated energetic penalty (Andersen and Koeppe, 2007; Killian, 1998). According to this model, proteins with longer transmembrane spans preferentially partition to the thicker cholesterol-rich trans Golgi, from which they are subsequently directed to the PM, while proteins with shorter transmembrane spans are retained in the earlier, thinner Golgi membranes (Munro, 1995; Sharpe et al., 2010).

Multiple experimental approaches have established a clear relationship between lipid bilayer thickness and its composition. Small-angle neutron scattering (SANS) studies have characterized the thicknesses of both the hydrophobic cores and hydrated layers of vesicles with various lipid compositions, demonstrating that hydrophobic core thickness increases with acyl chain length, while the water layer thickness remains relatively constant at ∼1.5–1.8 nm (Kucerka et al., 2009; Woodka et al., 2012). Small-angle X-ray scattering (SAXS) analyses have similarly demonstrated a linear relationship between hydrophobic core thickness and acyl chain length in phosphatidylcholine vesicles (Lewis and Engelman, 1983).

More recently, cryo-electron microscopy (cryo-EM) has enabled direct visualization of membrane thickness variations at high resolution (Cornell et al., 2020; Heberle et al., 2020; Schönnenbeck et al., 2025). The thicknesses of vesicles with defined lipid compositions have been measured as the distance between the two minima in line profiles of electron-scattering intensity across the lipid bilayer, obtained from 2D micrograph (Heberle et al., 2020; Schönnenbeck et al., 2025) and tomogram (Cornell et al., 2020) projections. These measurements confirm the linear relationship between acyl chain length and bilayer thickness. The minima-to-minima distances reported in the cryo-EM studies align well with the dimensions of the hydrophobic core measured by SANS and SAXS (Kucerka et al., 2009; Lewis and Engelman, 1983; Woodka et al., 2012). Although cryo-EM provides direct visualization of membranes at high resolution, it has primarily been applied to synthetic bilayers with reduced compositional complexity compared with native cellular membranes. Furthermore, existing approaches typically employ 2D analysis methods that do not account for variations in membrane curvature and, when relying on manual tracing and measurement, have limited throughput.

The converging evidence for organelle-specific lipid distributions and the well-documented relationship between lipid composition and membrane thickness suggest that cellular membranes may exhibit characteristic thickness profiles related to their functions. However, membrane thickness variations within cellular environments have largely remained uncharacterized. To this aim, we developed a semiautomated computational workflow to systematically measure membrane thickness from cryo-electron tomogram segmentations of diverse cellular membranes. Our approach allows for 3D curvature-aware thickness measurements in a voxel-wise manner with automated filtering of the results based on the respective tomogram intensities. The resulting membrane thickness maps can be overlaid with the original tomogram or with other structural features to provide biological context. By applying this workflow to a large publicly available *Chlamydomonas reinhardtii* dataset (Kelley et al., 2024, *Preprint*), we identified consistent thickness differences not only between organelle membranes but also within the bounds of individual organelles. Measurements in a human cell line yielded similar results and showed that membrane characteristics are affected by acute changes in lipid composition.

## Results

### A computational workflow for in situ membrane thickness analysis

**Figure 1.**
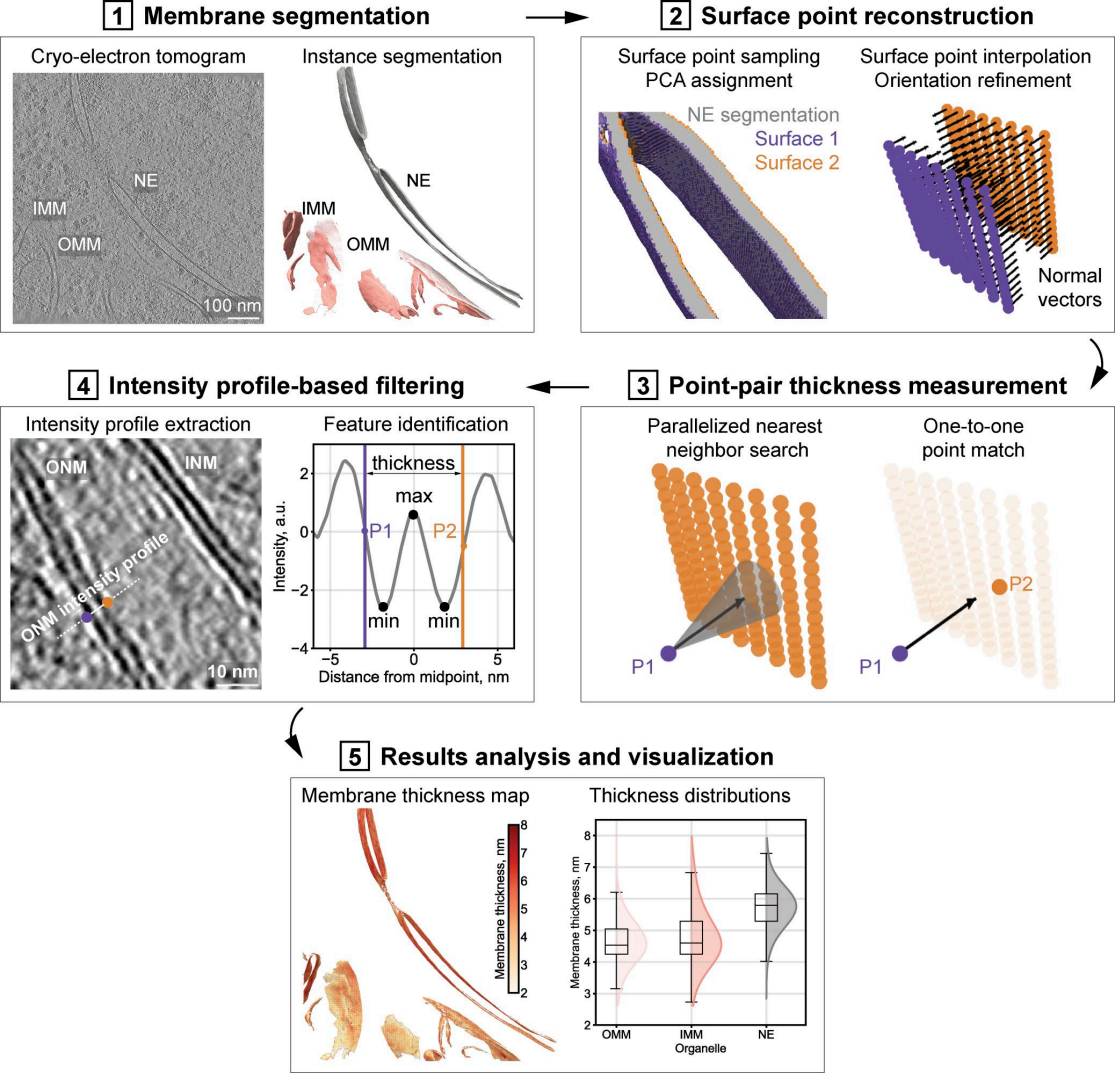
**Computational workflow for membrane thickness analysis from cryo-electron tomograms. (1)** Membrane segmentation. Reconstructed cryo-electron tomograms are processed with MemBrain-seg (Lamm et al., 2025, *Preprint*) to generate instance segmentations of different membranes in a given volume. **(2)** Surface point reconstruction. The coordinates and orientations of surface points are extracted with the marching cubes algorithm (Walt et al., 2014). A convolutional kernel is applied to retain only points lying on the segmentation boundaries. Surface point coverage can be increased through interpolation. Normal vectors (black arrows) are refined through local weighted neighbor averaging. The two surfaces are separated using principal component analysis (PCA) of normal orientations (surface points marked with purple and orange). **(3)** Point-pair thickness measurements. For each point on one surface, a ray is projected along its normal vector. A parallelized cone search identifies nearest neighbor candidates on the opposite surface. Points are paired one-to-one by a greedy search algorithm. Membrane thickness is calculated as the Euclidean distance between paired point coordinates. **(4)** Intensity profile-based filtering (recommended). Individual membrane intensity profiles are extracted for each thickness measurement by sampling the original tomogram in 3D along vectors extending from the coordinates of the paired points (in orange and purple). The intensity profile features can be used for automated filtering of the thickness measurements, e.g., by requiring that each profile have two minima positioned between (or close to) the paired measurement points (P1 and P2), the minima are separated by a central maximum, and they have a certain signal-to-noise ratio relative to the baseline. **(5)** Analysis and visualization of results. Output files include: .csv files with point coordinates, normal vectors, and surface assignments; .pkl files with intensity profiles for each point pair; .csv files with membrane thickness values (before and after filtering), including the coordinates of the paired points that generated each measurement. These latter files can be used to plot membrane thickness distributions or to output membrane thickness maps, where color intensity represents local membrane thickness (see tutorial).

Membrane thickness is a factor that directly influences the organization, structure, and function of membrane-associated proteins. Methods to reliably assess this parameter within a cellular context are therefore critical to understanding protein function at a molecular level. To date, cryo-electron tomography (cryo-ET) provides the highest-resolution direct visualization of native cellular structures within 3D volumes (Förster and Briegel, 2024). Membrane structures within cells exhibit variable morphologies and potentially heterogeneous thicknesses that may reflect differences in their biophysical or functional properties. To explore this question, we developed a computational workflow for semiautomated measurement of membrane thicknesses from membrane segmentations of cryo-ET data (Fig. 1). Our approach allows for quantitative assessment of thickness variations both within individual membranes and across organelles (or species), as well as for correlation of thickness with the coordinates of protein complexes in the same tomogram.

The computational workflow begins with instance segmentation of individual membrane entities within the tomogram (Fig. 1-1). We used MemBrain-seg with connected components analysis (Lamm et al., 2025, *Preprint*), though in principle other segmentation approaches can be applied. To improve segmentation accuracy, tomograms can be denoised (Buchholz et al., 2019) or filtered with a Wiener-like deconvolution filter (Tegunov and Cramer, 2019). During instance segmentation voxels containing different membrane structures are labeled with unique integer values. If needed, the segmentation labels can be manually curated (see Materials and methods) (Chiu et al., 2022; Lamm et al., 2025, *Preprint*; Wimmer, 2025).

**Figure 2.**
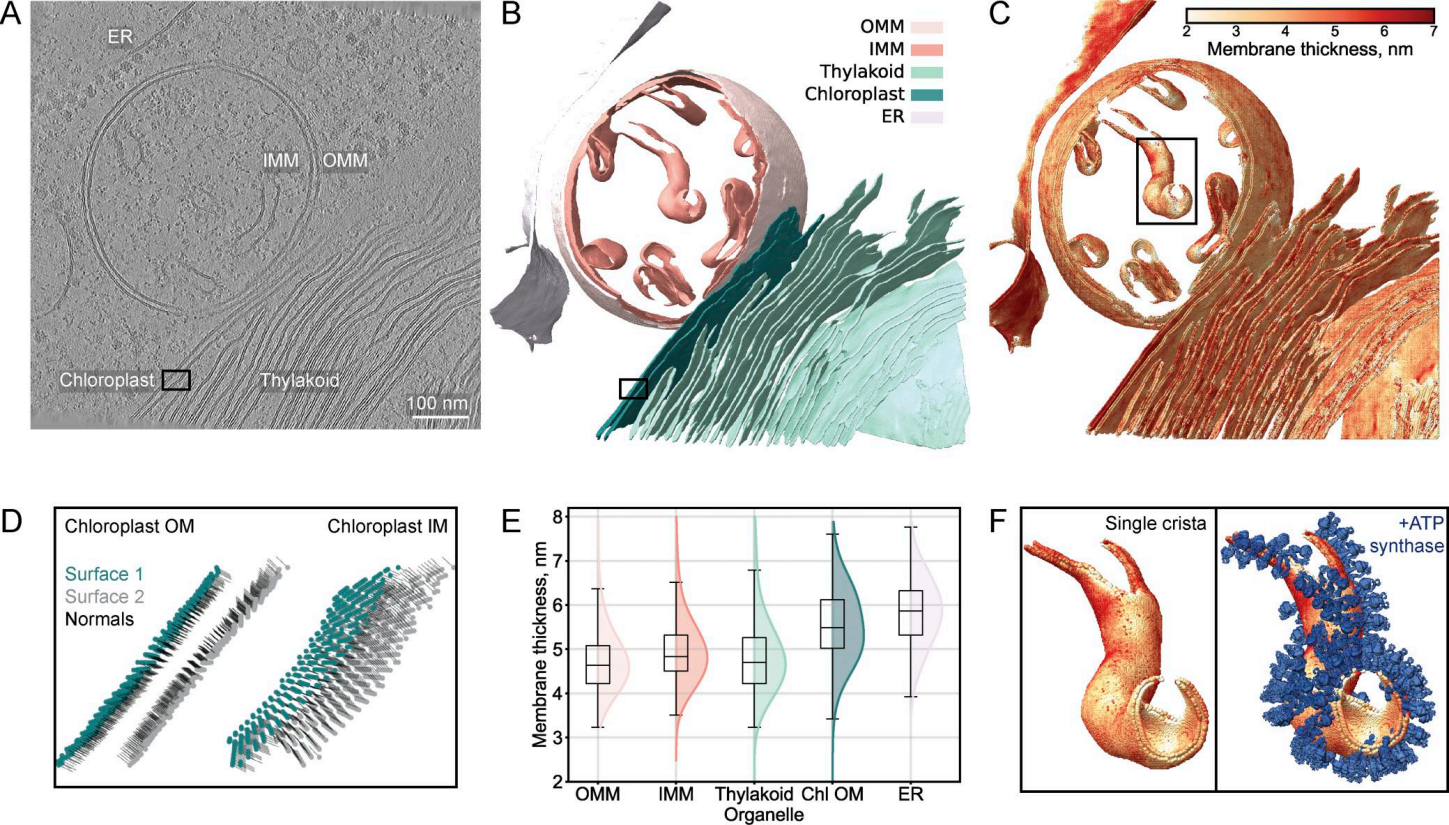
**Analysis of organelle membrane thickness heterogeneity within an exemplifying tomogram. (A)** Central slice from a denoised *C. reinhardtii* tomogram (Kelley et al., 2024, *Preprint*) (EMPIAR-11830, 2140.mrc, 7.84 Å/pix at bin4), showing the ER, IMM, OMM, thylakoid, and chloroplast membranes. Scale bar, 100 nm. **(B)** Instance segmentation generated by MemBrain-seg (Lamm et al., 2025, *Preprint*), with distinct colors marking different membrane instances. **(C)** Membrane thickness map, where color intensity represents differences in local membrane thickness. **(D)** Detailed view of the surface point extraction and normal vector orientation step, shown for a region of the chloroplast membranes (marked by black boxes in A and B). Points assigned to surface one in dark green, and to surface two in light gray. Black lines indicate normal vectors from each point to the opposite segmentation surface. **(E)** Comparative thickness distribution plots across membranes (color-coded as in B). Box plots show median (center line), interquartile range (box), and whiskers extending to 1.5× the interquartile range. **(F)** Co-localization of local membrane thickness with macromolecular complexes in a single mitochondrial crista (black box in C). ATP synthase particle positions were obtained from Righetto et al. (2025) as determined by subtomogram-averaging by Kelley et al. (2024, *Preprint*), with the resulting subtomogram average map (EMD-52802) used for visualization.

In the second step, the coordinates and orientations of surface points for a given labeled membrane instance are extracted from the segmentation volume (Fig. 1-2). Initial surface points are generated using the scikit-image implementation of the marching cubes algorithm (Walt et al., 2014), and a 3D convolution kernel is applied to retain only points lying on the segmentation boundaries. Additional surface points can be interpolated for comprehensive coverage. Each surface point is assigned an initial orientation, which is subsequently refined through weighted averaging to ensure smooth transitions across the surface, while still taking native membrane curvature into consideration. Surface points are assigned to the “inner” or “outer” membrane surface through a principal component analysis of the global normal vector orientations; this assignment is computational, as only the relative orientations and distances of surface points are relevant for the subsequent measurements.

**Figure 3.**
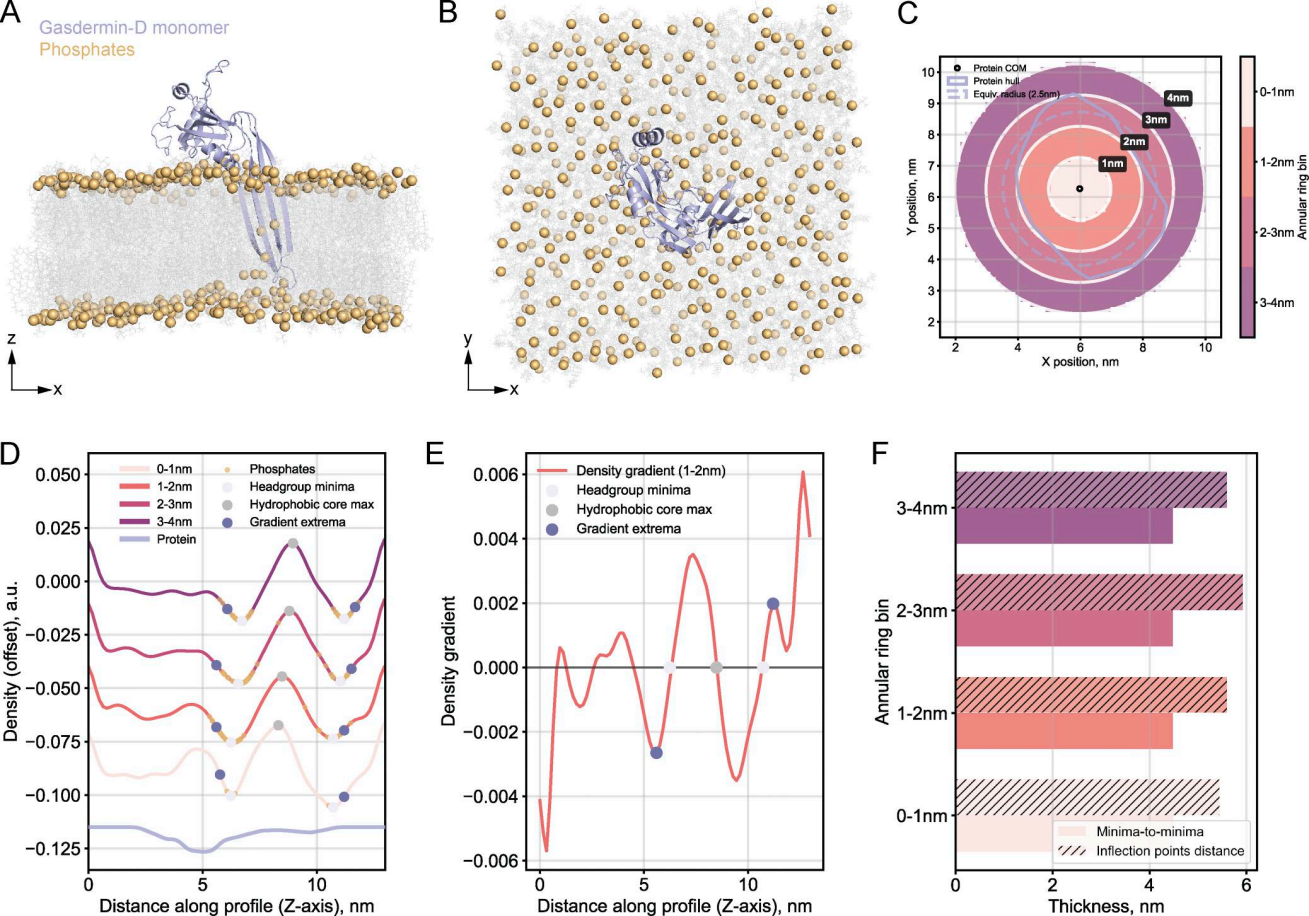
**Membrane protein effects on the density profiles and thickness measurements in MD-derived EM volumes. (A)** Side view (XZ) of gasdermin-D monomer (26.3 kDa, lilac) inserted into the extracellular leaflet of an asymmetric PM-like bilayer by Schaefer and Hummer (2022), converted to an EM density map (see Materials and methods). Lipid tails are shown as gray lines, and phosphate headgroups as yellow spheres. Water molecules are hidden for clarity. **(B)** Top view (XY) of the same simulation system. **(C)** Map of the distance-dependent analysis. Annotated: protein center of mass (COM, black point), convex hull footprint of the protein on the membrane (blue irregular outline), equivalent radius circle (dashed blue), and color-coded annular rings of extracted membrane regions at different distances from the protein center. **(D)** Density profiles per annular ring. Annotated: hydrophobic core maximum (gray point), headgroup density minima (light pink points), phosphate atom positions (yellow points), and profile inflection points (density gradient extrema, dark purple points). The isolated protein density profile is shown at the bottom for comparison. **(E)** Example density gradient profile (1–2 nm distance bin), where gradient values of zero correspond to headgroup minima and hydrophobic core maxima positions in the density profiles. Gradient extrema (inflection points on the profiles in D) are indicated by dark purple points. **(F)** Comparative membrane thickness measurements using minima-to-minima (light bars) distances vs the distances between inflection points (hatched bars, described in Materials and methods).

In the third step, membrane thickness is measured for each labeled instance in the volume (Fig. 1-3). The thickness estimation algorithm operates in two stages. First, each query point is processed in parallel by a dedicated GPU thread or CPU core. For each query point, the assigned thread sequentially searches all points on the opposite surface and identifies candidates that satisfy the following geometric constraints: (1) the target point falls within a 1° cone centered on the normal vector projected from the query point, and (2) the Euclidean distance between query and target points is below the user-specified maximum threshold. Each thread stores a predefined number of target candidates. Second, all candidates from the parallel GPU threads or CPU cores are aggregated into a global list, sorted by distance to the query point, and paired using a greedy search algorithm. Once paired, the query and target points are excluded from further consideration. Membrane thickness is calculated as the Euclidean distance between the coordinates of the paired points, with the normal-guided search accounting for the local 3D curvature.

**Figure 4.**
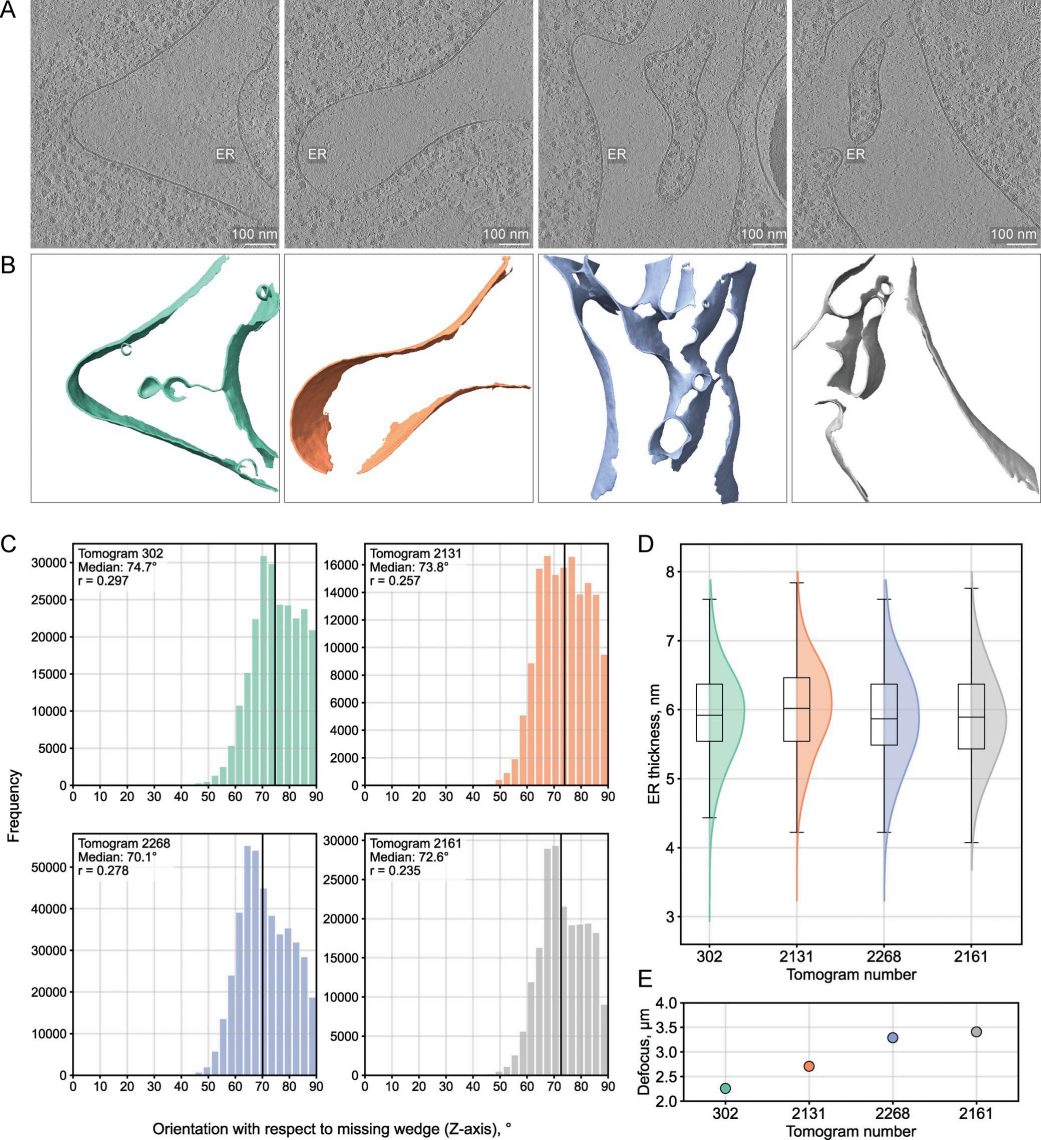
**Orientation-dependent analysis of membrane thickness measurements for the ER. (A)** Central slices from four representative of *C. reinhardtii* tomograms containing ER membranes in different orientations (Kelley et al., 2024, *Preprint*) (EMPIAR-11830, 7.84 Å/pixel at bin4, 302.mrc, 2131.mrc, 2268.mrc, and 2161.mrc). Scale bars, 100 nm. **(B)** MemBrain (Lamm et al., 2025, *Preprint*) segmentations of the corresponding ER membranes, color-coded to match the distribution plots in C. **(C)** Histogram distributions of membrane orientation angles relative to the missing wedge (0° = parallel, 90° = perpendicular to missing wedge), calculated using dot products between surface point normal vectors and the z-axis unit vector (see Materials and methods). The Pearson correlation coefficients between membrane thickness and orientation angle are indicated in each dataset’s legend. **(D)** Membrane thickness measurement distributions for the four ER instances. **(E)** Tomogram mean defocus values.

In the fourth step, intensity profiles are extracted and used to filter individual thickness measurements (Fig. 1-4). For each measurement, intensity profiles are extracted by sampling the original tomogram in 3D along vectors extending from the coordinates of the paired points. Characteristic membrane profiles should display two minima corresponding to the phosphate-rich headgroup regions, separated by a central maximum corresponding to the hydrophobic core (Falck et al., 2004). Detection of these profile features enables automated filtering of the thickness measurements. To ensure that uncertain measurements are excluded, two further requirements are imposed: minima must be positioned between the paired measurement points (or within a defined extension margin) and must have sufficient prominence above the local baseline to distinguish them from noise fluctuations. These filtering criteria are implemented as adjustable parameters within the pipeline to allow users to customize the thresholds.

**Figure 5.**
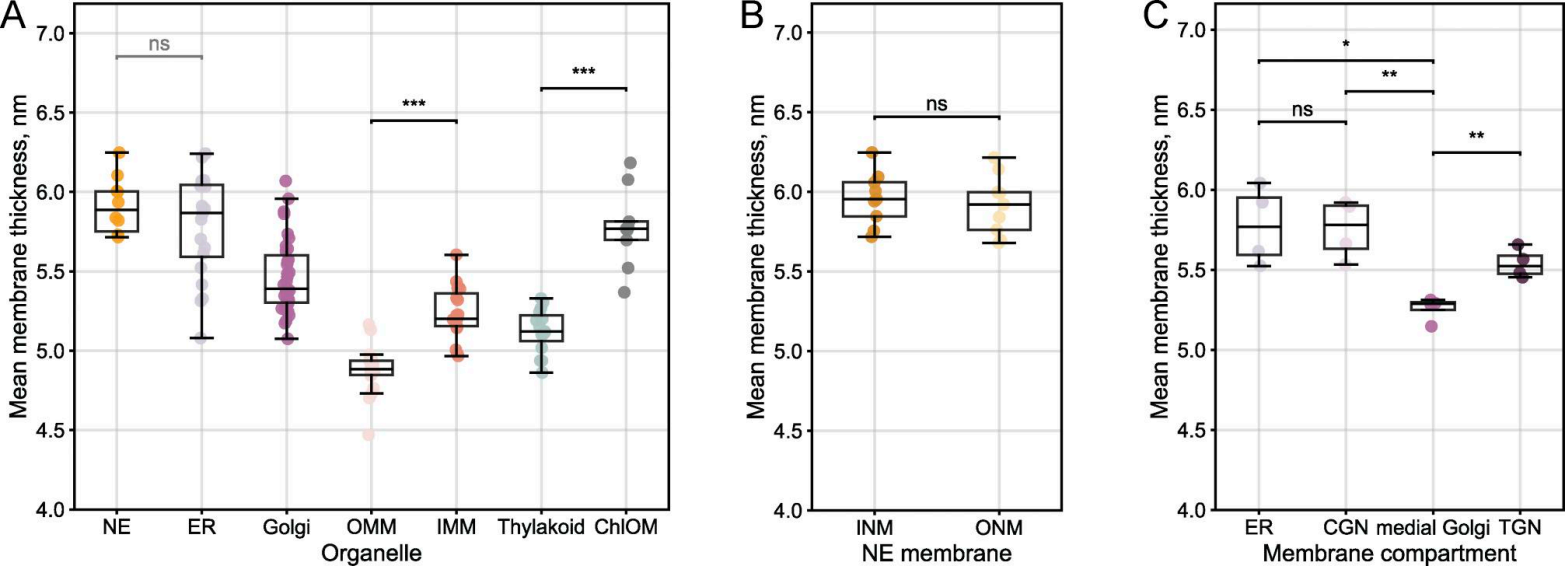
**Organelle-specific membrane thickness patterns across tomograms. (A)** Summary of mean membrane thicknesses across 51 tomograms from the *C. reinhardtii* dataset (Kelley et al., 2024, *Preprint*) (EMPIAR-11830). Box plots show median (center line), interquartile range (box), whiskers extending to 1.5× the interquartile range, and individual data points overlaid. Each point corresponds to the mean thickness value for a given membrane instance within a single tomogram (biological replicates): NE (analyzed as one continuous membrane, *n* = 10 instances, 2.2 M point-pair measurements), ER (*n* = 20 instances, 3.1 M point-pair measurements), OMM (*n* = 19 instances, 2.5 M point-pair measurements), IMM (*n* = 19 instances, 6.5 M point-pair measurements), thylakoid (*n* = 15 instances, 21.4 M point-pair measurements), and chloroplast outer membrane (Chl OM, *n* = 9 instances, 1.2 M point-pair measurements). For the Golgi apparatus, cisternae were analyzed individually and plotted as separate points (4 tomograms, 35 cisternae, 4.3 M point-pair measurements). Statistical comparisons of the mean values were performed for functionally related membranes: NE vs ER (two-sided unpaired *t* test, ns, P = 0.296), OMM vs IMM (two-sided paired *t* test, ***: P < 0.001), and thylakoid vs Chl OM (two-sided paired *t* test, ***: P < 0.001). **(B)** Mean membrane thickness comparison between inner and outer nuclear membranes (INMs and ONMs, respectively) for each NE instance. Two-sided paired *t* test: ns (P = 0.218). **(C)** Mean membrane thickness of ER and Golgi membranes, with Golgi cisternae classified as CGN, medial Golgi, and TGN based on manual assessment (*n* = 4 tomograms). Statistical comparisons using two-sided paired *t* tests reveal no significant difference between ER and CGN (ns: P = 0.732), significant differences between the ER and medial Golgi (*: P = 0.022) and CGN and medial Golgi (**: P = 0.007), and significant thickening from medial to TGN cisternae (**: P = 0.005).

Finally, the results are outputted in multiple formats for both qualitative and quantitative analysis (Fig. 1-5). The thickness measurement files can be used for statistical comparisons of thickness distributions across organelles or experimental conditions and to generate membrane thickness maps, where color intensity corresponds to local thickness variations. The coordinates of the paired points can be used for contextual analysis through co-localization with the coordinates of membrane proteins or other structures of interest. The visualization options allow for comprehensive analysis of membrane thickness in different cellular contexts (see tutorial).

### Membrane thickness measurements applied to in situ data

To test the performance of our workflow on in situ data, we applied it to a publicly available denoised tomogram of the green algae *C. reinhardtii* (Kelley et al., 2024, *Preprint*) (Fig. 2 A).

Using MemBrain-seg’s connected component analysis (Lamm et al., 2025, *Preprint*), we generated an instance segmentation volume where distinct voxel values corresponded to different organelle membrane entities, including the rough endoplasmic reticulum (ER), inner and outer mitochondrial membranes (IMMs and OMMs), thylakoid, and chloroplast envelope membranes (Fig. 2 B). From this segmentation, we computed a membrane thickness map (Fig. 2 C). We extracted the coordinates and orientations of surface points and classified them into opposing surfaces, as demonstrated for a section of the chloroplast membranes, where the normal vectors point toward the opposite segmentation surface (Fig. 2 D).

**Figure S1.**
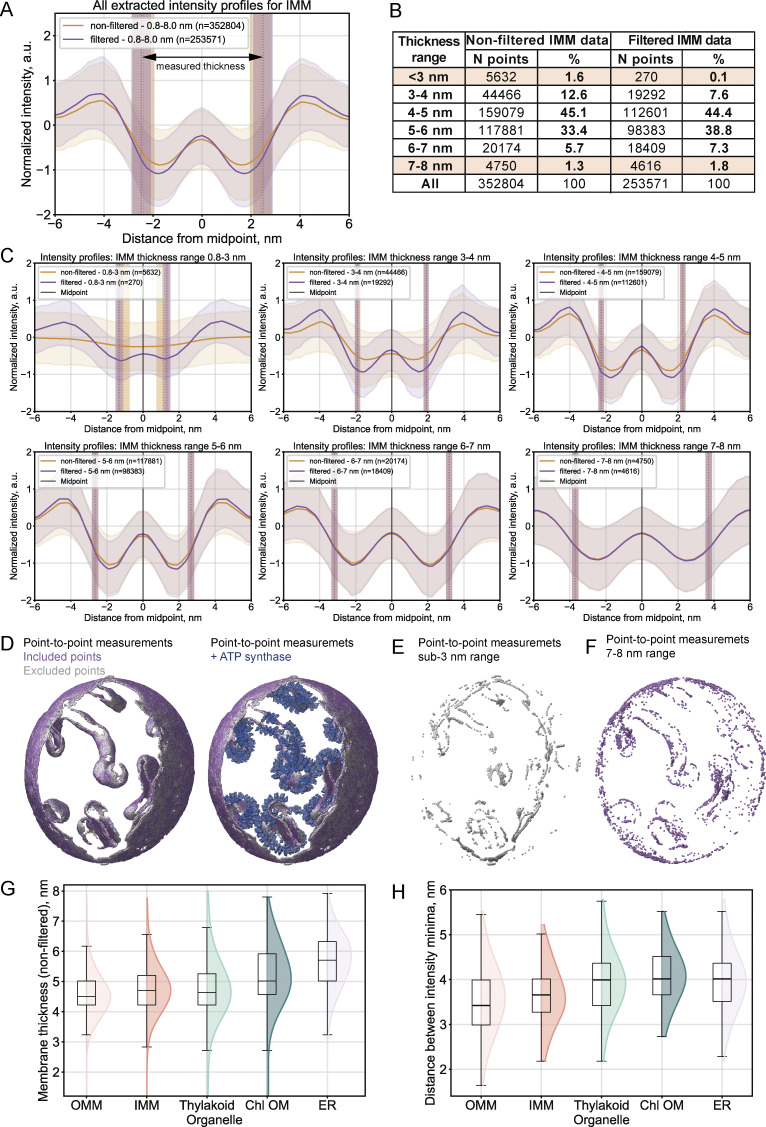
**Intensity profile-based filtering of the IMM thickness measurements from** Fig. 2 **. (A)** Aggregated intensity profiles for all IMM thickness measurements. The mean profiles prior to filtering are shown in orange, those after filtering in purple, with standard deviations indicated by shading. Membrane thickness is measured between paired points on opposing membrane segmentation surfaces, with their mean positions indicated by vertical lines and standard deviations shown as shaded regions. These measurement positions correspond to the segmentation boundaries. Filtering criteria: two identifiable minima separated by a central maximum; the minima are positioned between the paired points or within a 30% extension range outward (≈1–1.5 voxels extension in bin4 tomograms). **(B)** Summary statistics for the number of measurements before and after filtering across multiple thickness bins. **(C)** Intensity profiles binned by thickness ranges (before filtering in orange, after filtering in purple). **(D)** Spatial distribution of the filtering results. Left panel: Included measurement points are colored in purple; excluded points are shown in gray. Right panel: ATP synthase coordinates (blue) are overlaid for context. ATP synthase particle positions were obtained from a publicly available repository (Righetto et al., 2025) as determined by subtomogram averaging by Kelley et al. (2024, *Preprint*), with the resulting subtomogram average map (EMD-52802) used for visualization. **(E and F)** Spatial distribution of filtering results for the sub-3 nm (gray) and 7–8 nm (purple) thickness ranges, respectively. **(G)** Membrane thickness distributions prior to filtering across all measured organelle membranes for direct comparison with Fig. 2 E. **(H)** Minima-to-minima distances measured from tomogram-derived intensity profiles of each organelle shown in G.

All thickness measurements were filtered based on features of the extracted intensity profiles (see above, mean IMM profiles prior to and after filtering are plotted in Fig. S1 A). Membrane thickness was measured between paired segmentation boundary points, projected as vertical lines on the slopes of the extracted profiles. While the filtering procedure primarily excluded measurements in the sub-3 nm range, these extreme values represented a small subset of all measurements, as >97% of results fell in the 3–7 nm range (Fig. S1, B and C). Extreme thickness measurements often coincided with highly curved membrane regions; however, we cannot exclude that other factors contributed to the observed spatial patterns (Fig. S1, D–F). We considered the retained measurements as “valid” if they passed the filtering criteria.

Hundreds of thousands of individual filtered distance measurements per membrane instance yielded highly representative thickness distributions (Fig. 2 E). We observed clear thickness differences between organelle membranes within the same tomogram, where projection images were acquired under identical imaging conditions. For functionally related membranes, the IMM showed higher thickness values compared with the OMM, and the outer chloroplast membrane (Chl OM) was thicker than the thylakoid. These thickness differences were not artifacts of the filtering procedure, as the same trends could be observed in the non-filtered data (Fig. S1 G). The thickness patterns were similarly preserved when membrane thickness was defined as the distance between intensity profile minima (Fig. S1 H), but the absolute values differed. We explore the implications of measurement point positioning along the intensity profiles in the subsequent section.

To demonstrate the potential for integrating thickness measurements with protein localization data, we overlaid the thickness maps with the coordinates of ATP synthase complexes, as determined through subtomogram averaging by Kelley et al. (2024, *Preprint*) (Fig. 2 F and Video 1). Quantitative spatial correlation can be used to reveal relationships between membrane thickness, curvature, and the structural organization of membrane proteins.

**Video 1.** **Detailed visualization of the membrane thickness map in** Fig. 2 C **with overlaid coordinates of ATP synthase (blue) (** Kelley et al., 2024 **, *Preprint*), presented in** Fig. 2 F **.** The video includes zoomed-in views and rotations of specific regions of the model. Membrane thickness is color-coded according to the scale in Fig. 2 C, with lighter colors indicating thinner membrane regions and darker reds representing thicker areas.

### How proteins affect membrane thickness measurements

Cellular membranes are composite structures containing numerous proteins that might affect thickness measurements. Protein localization in cryo-ET is laborious and currently feasible only for large or abundant complexes, leaving smaller proteins below single-tomogram resolution limits unaccounted for. To circumvent these experimental limitations, we used the full atomic representations from published molecular dynamics (MD) simulations and converted them to resolution-limited EM density maps for thickness analysis (see Materials and methods).

We generated an EM volume of the monomeric gasdermin-D (26.3 kDa) embedded in an asymmetric PM-like bilayer (Schaefer and Hummer, 2022) (Fig. 3, A and B) and extracted density profiles in annular rings at various distances from the protein center of mass (Fig. 3 C). Even within 1 nm of the protein center, we could identify two clear minima and a maximum in the profiles (Fig. 3 D). This highly contrastive electron optical density reflects the strong electron-scattering properties of the phosphate headgroups and their lateral concentration within the membrane plane, which dominates the electron density contribution from the protein atoms.

When projected onto the density profiles, the phosphates (yellow points in Fig. 3 D) have Gaussian-like distributions centered near the profile minima but extending outward on the slopes. To identify the membrane interface boundaries, we analyzed the density gradients, which capture the rate of density change (Fig. 3 E). The gradient equals zero at density minima and maxima, while the gradient extrema mark the positions of steepest density change (the inflection points on the profiles). We identified the first gradient extrema extending outward from the hydrophilic headgroup minima. These inflection points mark approximately the outer edge of the phosphate distributions on the density profiles (Fig. 3 D) and broadly align with the segmentation boundaries from experimental tomograms (Fig. S1 A). We quantified membrane thickness from either minima-to-minima or inflection point distances (Fig. 3 F). Thickness values were consistent at all distances from the protein center, indicating that the presence of the protein does not interfere with thickness measurements.

**Figure S2.**
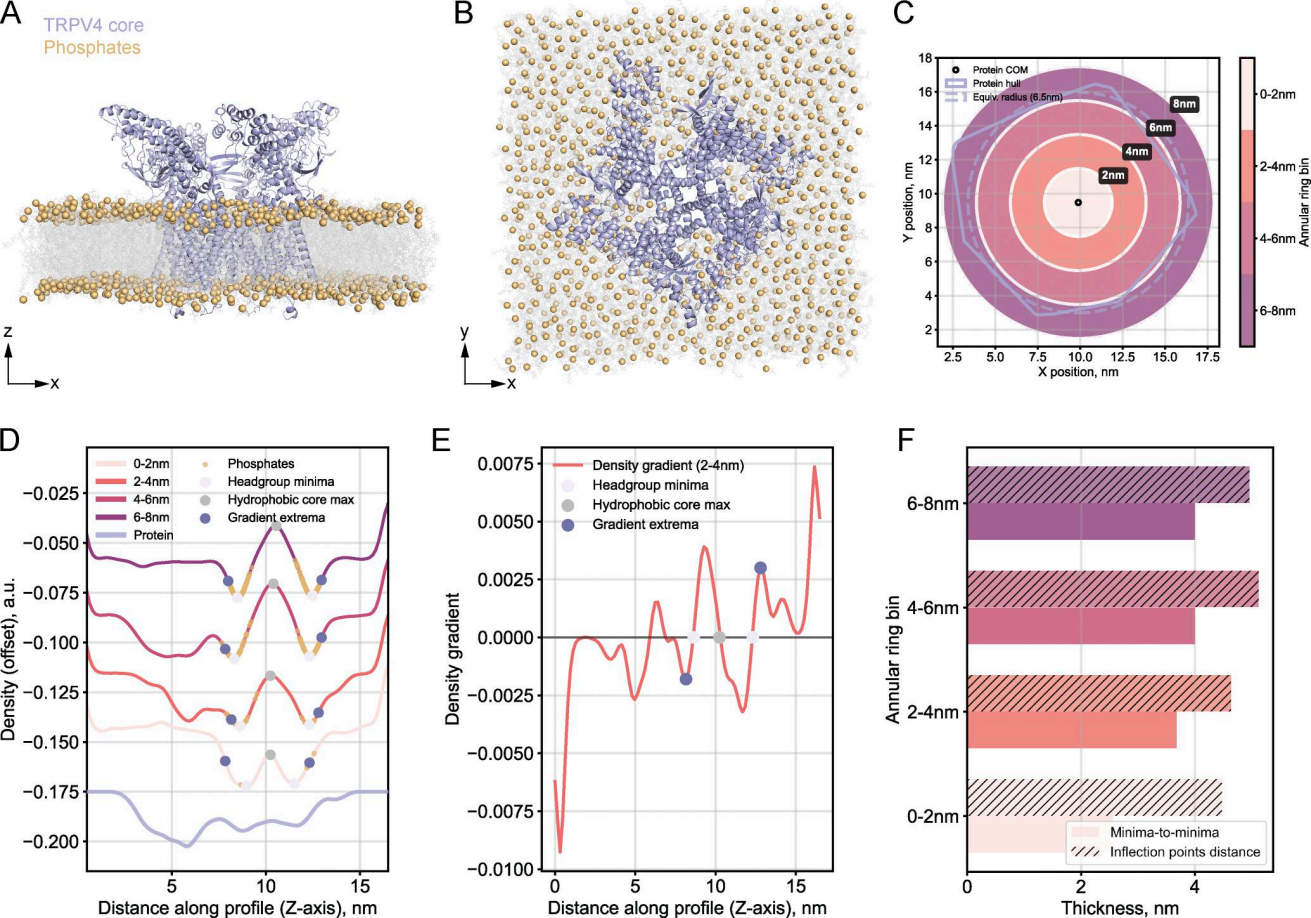
**Effects of a larger membrane protein on the density profiles and thickness measurements in MD-derived EM volumes. (A)** Side view (XZ) of a tetrameric TRPV4 core (281.6 kDa, lilac) in a symmetric phosphatidylcholine (POPC) bilayer by Goretzki et al. (2023), converted to an EM density map (see Materials and methods). Lipid tails are shown as gray lines, and phosphate headgroups as yellow spheres. Water molecules are hidden for clarity. **(B)** Top view (XY) of the same simulation system. **(C)** Map of the distance-dependent analysis. Annotated: protein center of mass (COM, black point), convex hull footprint of the protein on the membrane (blue irregular outline), equivalent radius circle (dashed blue), and color-coded annular rings of extracted membrane regions at different distances from the protein center. **(D)** Density profiles per annular ring. Annotated: hydrophobic core maximum (gray point), headgroup density minima (light pink points), phosphate atom positions (yellow points), and profile inflection points (density gradient extrema, dark purple points). The isolated protein density profile is shown at the bottom for comparison. **(E)** Example density gradient profile (1–2 nm distance bin). Gradient extrema (inflection points on the profiles in D) are indicated by dark purple points. **(F)** Comparative membrane thickness measurements using minima-to-minima (light bars) distances vs distances between inflection points (hatched bars, described in Materials and methods). Thickness measured by both approaches is smaller compared with the simulated asymmetric PM-like system in Fig. 3 F.

To assess whether a larger protein complex might distort the density profiles, we analyzed tetrameric TRPV4 (281.6 kDa) embedded in a symmetric phosphatidylcholine bilayer (Goretzki et al., 2023) (Fig. S2). Membrane thickness remained consistent, with only a minor reduction in the minima-to-minima distance in the immediate vicinity of the protein (Fig. S2 F). Importantly, any protein-induced effects on the density profiles occurred at distances comparable with typical experimental tomogram resolutions (c.a. 1 nm/voxel for bin4 data), suggesting that such perturbations might be hard to detect or masked by artifacts during cryo-ET data acquisition and processing.

### Membrane orientation does not strongly affect thickness measurement

**Figure S3.**
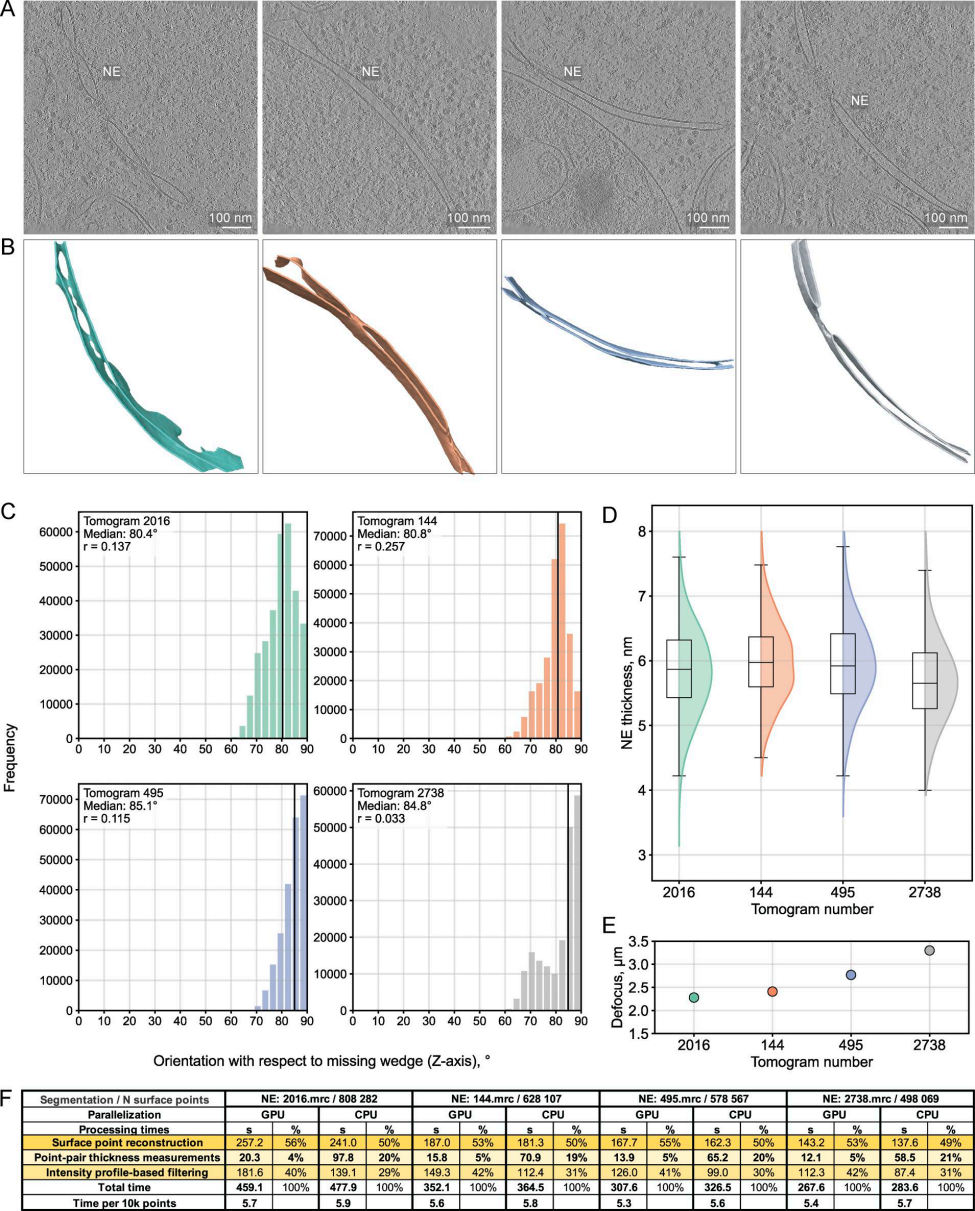
**Orientation-dependent analysis of membrane thickness measurements for the NE. (A)** Central slices from four representative *C. reinhardtii* tomograms displaying NE membranes in different orientations (Kelley et al., 2024, *Preprint*) (EMPIAR-11830, 7.84 Å/pixel at bin4, 2016.mrc, 144.mrc, 495.mrc, and 2738.mrc). Scale bars, 100 nm. **(B)** MemBrain (Lamm et al., 2025, *Preprint*) segmentations of the corresponding NE membranes, color-coded to match the distribution plots in C. **(C)** Histogram distributions of membrane orientation angles relative to the missing wedge (0° = parallel, 90° = perpendicular to missing wedge). The Pearson correlation coefficients are indicated in each legend. **(D)** Membrane thickness measurement distributions for the four NE instances. Box plots show median (center line), interquartile range (box), and whiskers extending to 1.5× the interquartile range. **(E)** Mean defocus values per tomogram. **(F)** Compute times per processing step for each NE segmentation. Point-pair thickness measurements are parallelized on either NVIDIA A100 GPU (Linux system, 4–5x faster execution) or on four MacOS CPU cores. Surface point reconstruction and intensity profile filtering run on single CPU cores with comparable performance across systems.

The missing wedge artifact in cryo-ET leads to signal elongation, predominantly affecting densities oriented parallel to the missing wedge axis (usually the z axis of the reconstructed tomogram). Using four ER instances as a test case, we calculated membrane normal vector orientations with respect to the z axis. We computed Pearson correlation coefficients between the orientation angles and thickness measurements for all valid paired points (Fig. 4). Correlation coefficients remained consistently small (<0.3, Fig. 4 C), indicating a minimal orientation-dependent bias in the thickness results even for a convoluted membrane such as the ER. In addition, we found no apparent relationship between tomogram defocus values and ER thickness (Fig. 4 E). An identical analysis on four nuclear envelope (NE) instances, predominantly oriented perpendicular to the missing wedge, produced even weaker correlations (Fig. S3). We report computing times for processing the NE segmentations in Fig. S3 F as examples of the pipeline runtime on representative experimental data.

### Relative membrane thicknesses are consistent across *Chlamydomonas* organelles

To investigate whether the organelle-specific membrane thickness patterns observed in a single tomogram (Fig. 2) represent consistent, potentially biologically relevant features, we extended the analysis to 51 denoised tomograms from the EM Public Image Archive (EMPIAR)-11830 *Chlamydomonas* dataset (Kelley et al., 2024, *Preprint*). For each membrane of interest, we analyzed individual instances in tomograms from different acquisition sessions: NE (*n* = 10), ER (*n* = 20), OMMs and IMMs (*n* = 19 each), Golgi apparatus (*n* = 4), thylakoid (*n* = 15), and outer Chl OMs (*n* = 9). Using instance segmentations with unique membrane labels as inputs, we generated millions of point-to-point distance (thickness) measurements per membrane type.

To compare thickness consistency across tomograms, we plotted the mean thickness values of each membrane type within each analyzed tomogram (Fig. 5 A). The Golgi represents a special case, where we plotted the mean thickness of each individual cisterna to capture the thickness variations across the stack. We found that NE and ER membranes exhibited the highest mean thickness values (5.1–6.3 nm), followed by Chl OM (5.4–6.2 nm), the Golgi (5.1–6.1 nm), the thylakoid (4.9–5.4 nm), and mitochondrial membranes (OMM: 4.5–5.2 nm, IMM: 5.0–5.6 nm). Statistical analysis revealed consistent patterns across tomograms: while NE and ER membranes had similar mean thicknesses (unpaired *t* test, ns), paired *t* tests on a per-tomogram basis showed statistically significant differences in the mean thicknesses between the OMM and IMM (P < 0.001), the thylakoid and Chl OM (P < 0.001). In contrast to mitochondria and chloroplasts, we found no statistically significant differences between the thickness of inner and outer NE membranes (paired *t* test, Fig. 5 B).

We manually assigned each cisterna in the Golgi stack to the cis-Golgi network (CGN), medial Golgi, or trans-Golgi network (TGN) and plotted their mean thickness values alongside adjacent ER membranes (Fig. 5 C). CGN and ER membranes showed similar thickness values, while medial Golgi membranes displayed the lowest thickness values within the stack. Moving toward the trans face, we observed a statistically significant increase in membrane thickness. Although we report paired *t* test results in the figure, we advise against their overinterpretation due to the small number of analyzed Golgi instances. Instead, we would like to highlight the pattern of thickening from medial to TGN (see below).

### Relative membrane thicknesses are conserved across human organelles

**Figure 6.**
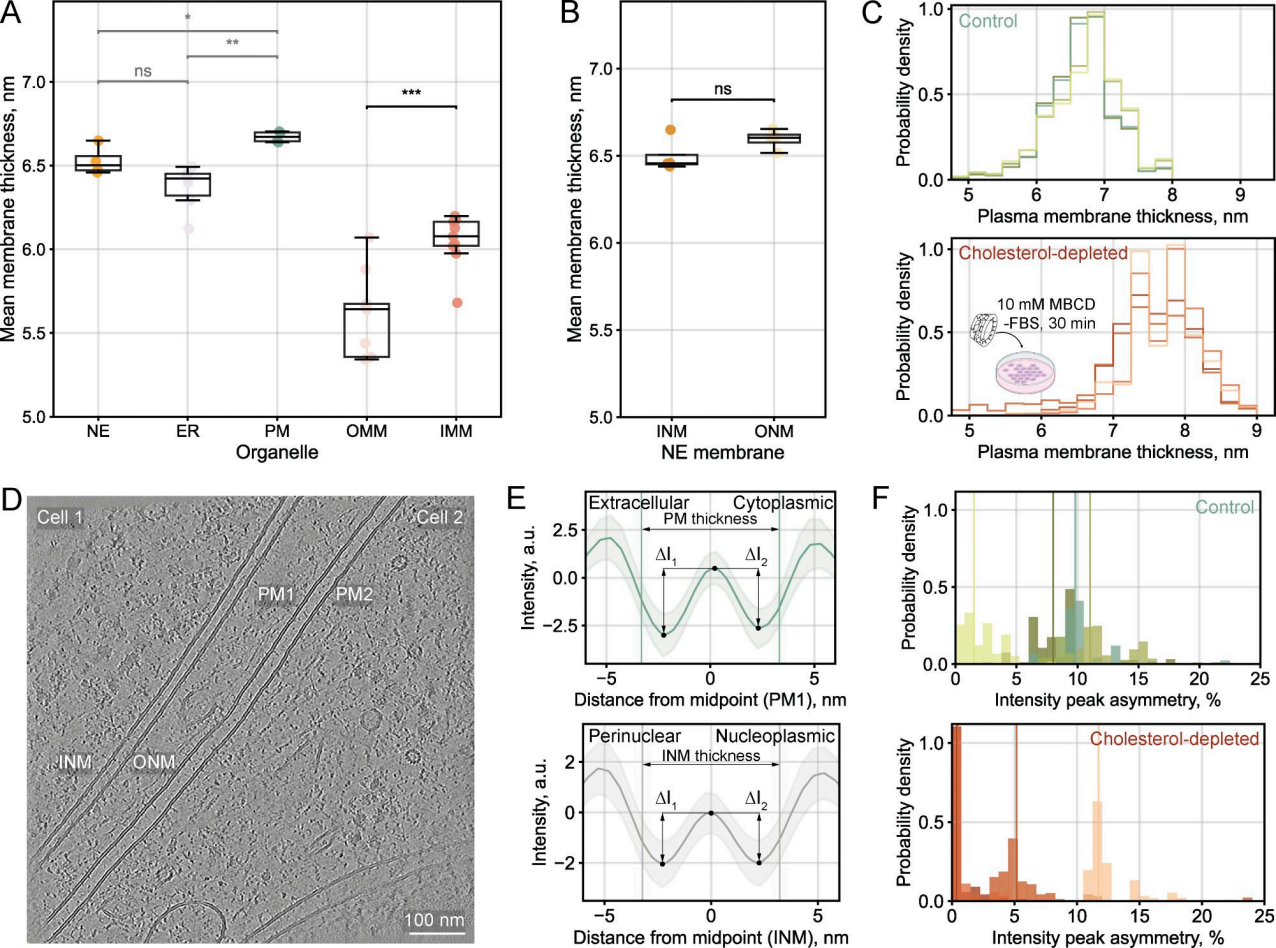
**Organelle-specific thickness patterns in human cells, including leaflet asymmetry analysis. (A)** Summary of mean membrane thicknesses across organelles in human embryonic kidney cells (HEK293). Box plots show median (center line), interquartile range (box), whiskers extending to 1.5× the interquartile range, and individual data points overlaid. Each point corresponds to the mean thickness value for a given membrane instance: NE (analyzed as one continuous membrane, *n* = 4 instances, 0.7 M point-pair measurements), ER (*n* = 6 instances, 0.4 M point-pair measurements), PM (*n* = 4 instances, 0.5 M point-pair measurements), OMM (*n* = 9 instances, 0.8 M point pair measurements), and IMM (*n* = 9 instances, 3.2 M point-pair measurements). Statistical comparisons of mean thickness values: NE vs ER (two-sided unpaired *t* test, ns: P = 0.076), NE vs PM (two-sided unpaired *t* test, *: P = 0.021), ER vs PM (two-sided unpaired *t* test, **: P = 0.003), and OMM vs IMM (two-sided paired *t* test, ***: P < 0.001). **(B)** Comparison of inner versus outer nuclear membrane (INM and ONM) thicknesses for individual NE instances. Two-sided paired *t* tests of the mean INM and ONM thicknesses: ns (P = 0.070). **(C)** Validation of the sensitivity of the thickness measurement algorithm using cholesterol depletion in HEK293 cells with MBCD (10 mM, 30 min). Normalized thickness distributions of PM instances from control (*n* = 4) and cholesterol-depleted (*n* = 4) cells. **(D)** Central slice from a tomogram showing two control HEK293 cells in contact, with two PMs (PM1 and PM2) at the cell–cell interface and NE membranes (INM, ONM) in cell 1. Scale bar, 100 nm. **(E)** Mean intensity profiles for PM1 (upper) and INM (lower), with intensity minima marking the headgroup regions and central maxima—the hydrophobic cores. Using the coordinates of the paired measurement points, one can trace back to the original tomogram to determine which cellular compartment each headgroup minima faces—the PM leaflet can be on the extracellular or cytoplasmic side; the INM leaflet—on the perinuclear or the nucleoplasmic side. Vertical lines mark the mean positions of paired measurement points used for thickness calculation. The asymmetry score is calculated as the ratio of the intensity value differences (ΔI_1_ and ΔI_2_) between each headgroup minimum and the central maximum, with the larger Δ in the nominator. To reduce noise from individual measurements, we binned and averaged the intensity profiles based on membrane thickness (see Materials and methods). **(F)** Thickness-binned asymmetry distributions for four control (green) and four cholesterol-depleted (red) PMs, with median values marked by vertical lines. Perfect symmetry corresponds to a score of 1.0 (or 0% asymmetry), while increasing asymmetry yields asymmetry scores >1.0, with percentage asymmetry derived as (score -1.0) × 100% (e.g., 1.05 = 5% asymmetry).

To further validate our workflow and to determine whether the organelle-specific thickness patterns observed for *Chlamydomonas* represent a more general principle of membrane biology, we acquired a smaller tomographic dataset of human embryonic kidney (HEK293) cells. These tomograms were processed using the described workflow. While there were small variations in the absolute thickness values between human and *Chlamydomonas* cells, we observed similar relative thickness relationships between different membrane types and organelles (Fig. 6 A). The PM was the thickest measured membrane in these cells. The NE and ER were consistently thicker than the mitochondrial membranes. Within mitochondria, the IMM was consistently and significantly thicker than the OMM (paired *t* test, P < 0.001), in line with the relationship observed for *Chlamydomonas*. We found no statistically significant difference between the thicknesses of the inner and outer NE membranes (Fig. 6 B).

These findings demonstrate two important points: first, the computational workflow can be applied to tomograms containing different membrane types across species, and second, relationships between the thicknesses of certain membranes appear to be conserved across human and algae cells. The general nature of these thickness patterns across evolutionarily distant species may suggest functional requirements that dictate similar distributions of lipids and membrane proteins across cellular compartments.

**Figure S4.**
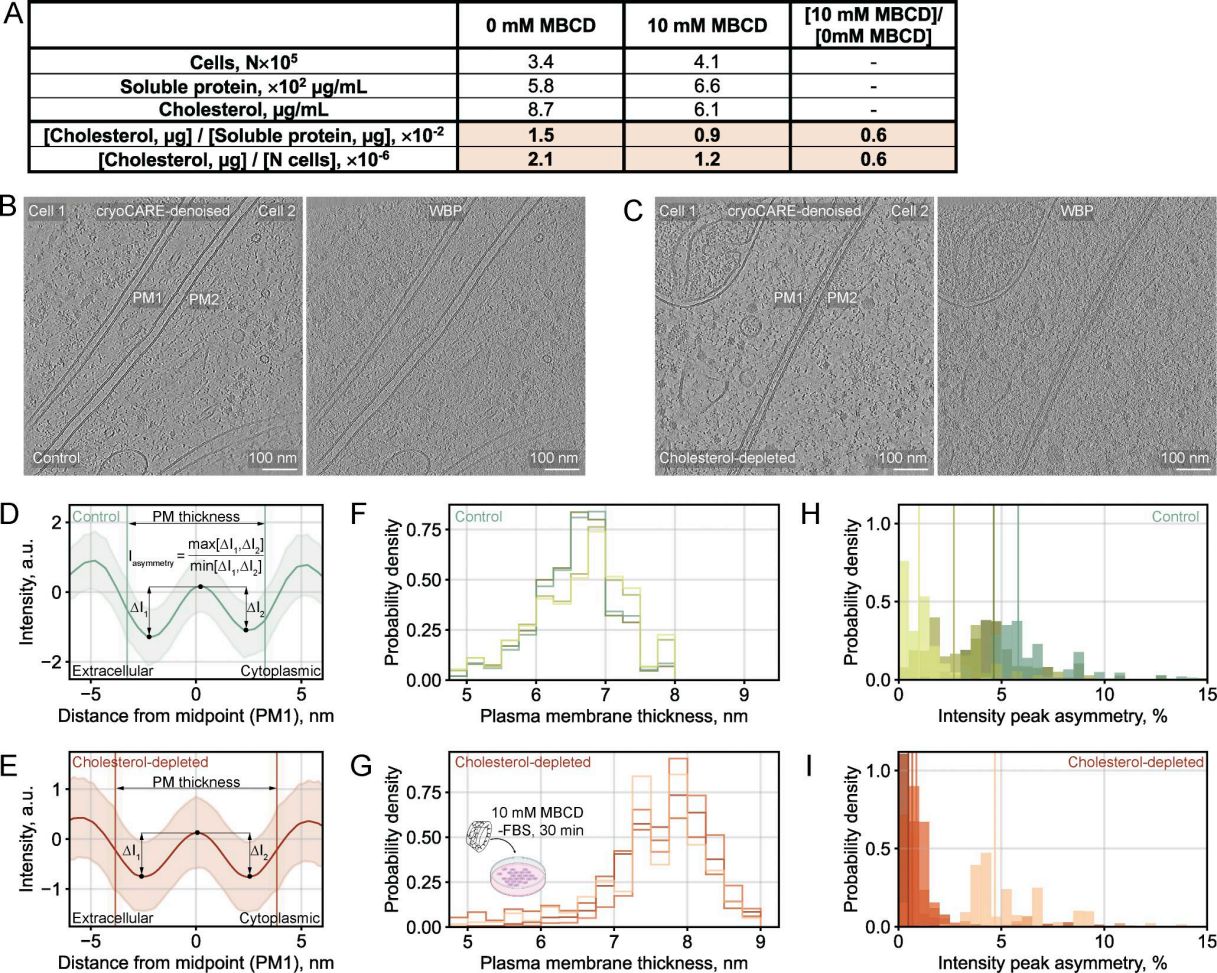
**Comparison of thickness and asymmetry measurements across tomogram reconstruction methods for control vs cholesterol-depleted cells. (A)** Fluorometric assay results for validation of cholesterol depletion (see Materials and methods). The table shows total cell counts, soluble protein concentration, and cholesterol levels in each condition, along with normalized values (cholesterol per protein [µg/µg] and µg cholesterol per cell). When normalized to protein content or per cell, treatment with 10 mM MBCD reduced cholesterol levels by 40%. **(B and C)** Central slices from tomograms reconstructed with WBP algorithm from cryoCARE denoised (left) or unprocessed frames (right), showing control (B) and cholesterol-depleted (C) HEK293 cells in contact. Two PMs (PM1, PM2) are visible at the cell–cell interfaces. The cryoCARE-denoised tomogram shown in B is the same as in Fig. 6 D, shown for compa. Scale bars, 100 nm. **(D and E)** Mean intensity profiles for PM1 extracted from WBP tomograms from unprocessed frames in control (D) and cholesterol-depleted (E) cells. Vertical lines mark the mean positions of paired measurement points used for thickness calculation. For details on the asymmetry score calculation, see Materials and methods. **(F and G)** Normalized thickness distributions from WBP tomograms from unprocessed frames for control (F) and cholesterol-depleted (G) cells. **(H and I)** Thickness-binned asymmetry distributions for control (H) and cholesterol-depleted (I) PMs derived from WBP tomograms, expressed as percentage asymmetry where 1.0 = 0% asymmetry (or perfect symmetry) and values >1.0 indicate increasing asymmetry [(score -1.0) × 100%].

We hypothesized that the consistent membrane thickness relationships might in part reflect their different lipid compositions, as suggested by lipidomic analysis. To test whether our approach could detect lipid composition-dependent changes in membrane characteristics, we performed a pharmacologic perturbation experiment using methyl-β-cyclodextrin (MBCD), a compound that acutely extracts cholesterol from the PMs of cells (Mahammad and Parmryd, 2015; Rodal et al., 1999). We acquired tilt-series on thinned lamellae targeting the PM in MBCD-treated and control cells and assessed the effectiveness of cholesterol depletion in parallel using a fluorometric detection assay (Fig. S4 A).

Analysis of four PM instances per condition revealed a shift toward higher membrane thickness in cholesterol-depleted cells (Fig. 6 C). To probe for other changes to membrane organization, we analyzed PM leaflet asymmetry using the extracted intensity profiles (Fig. 6, D–F) and calculated asymmetry scores by comparing the relative depths of each leaflet’s minima with the central hydrophobic maximum, similarly to Heberle and Doktorova (2025). We grouped the profiles based on thickness and used their mean features to avoid confounding the results with individual noisy measurements. While both conditions showed variability (one outlier per condition), we observed a trend toward decreased PM leaflet asymmetry in cholesterol-depleted cells (Fig. 6 F). To assess if these findings could be an artifact of tomogram preprocessing, we repeated the analysis using weighted back projection (WBP)-reconstructed tomograms from unprocessed frames and observed identical trends of increased membrane thickness and reduced PM leaflet asymmetry for the cholesterol-depleted cells (Fig. S4, B–I). Although our limited number of instances prevents us from drawing definitive conclusions, these findings demonstrate that our workflow can detect subtle changes in membrane characteristics following perturbation experiments (see Discussion).

### Functionally distinct membranes within an organelle differ in thickness

The distinct functional roles of membranes within the same organelle are often reflected in their lipid and protein compositions, which likely account for the thickness variations we measured between the IMM and OMM in both *Chlamydomonas* and human cell tomograms (see Discussion).

**Figure 7.**
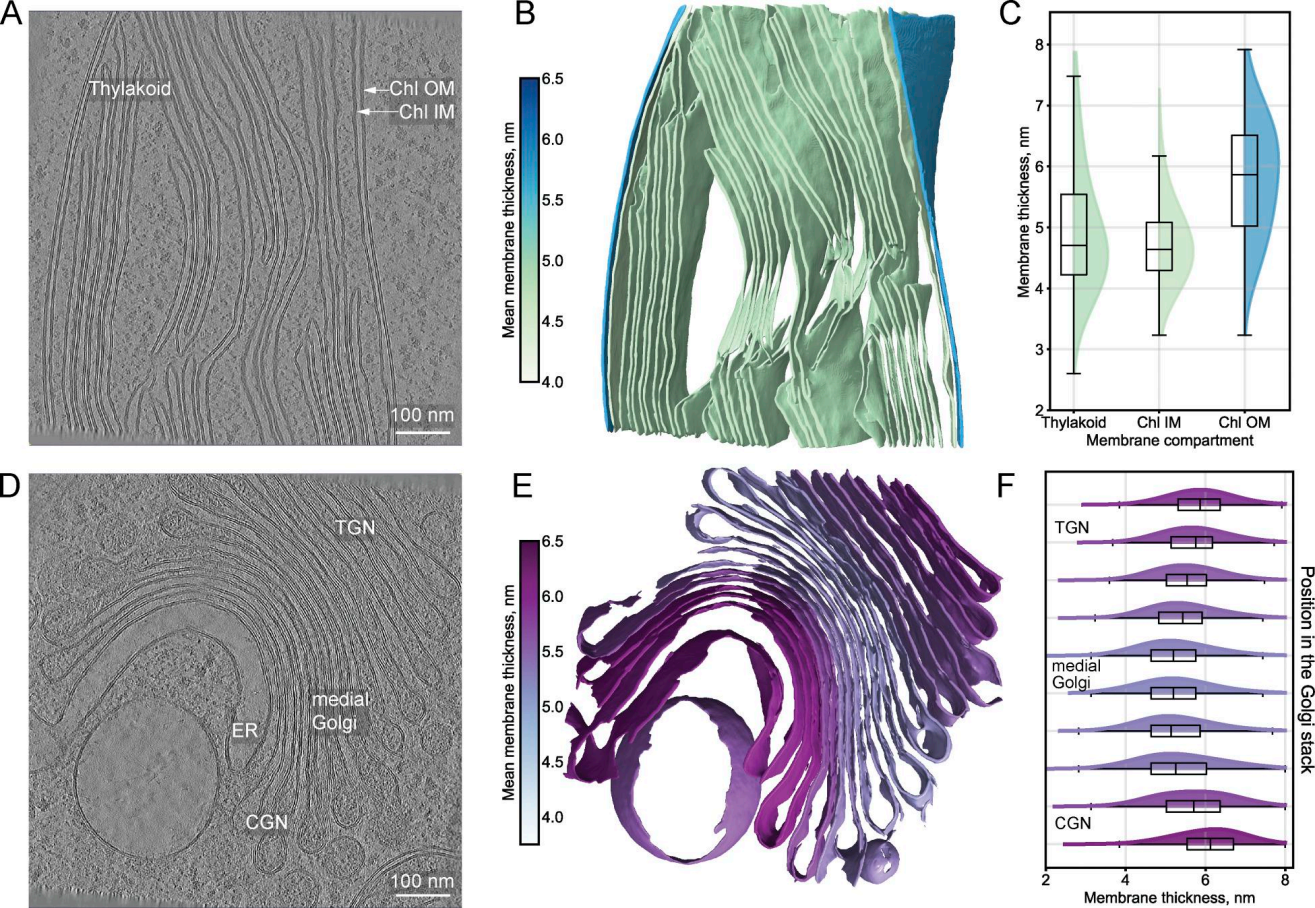
**Membrane thickness variations between functionally distinct membranes within the same organelle. (A–C)** An analysis of the chloroplast membrane system. **(A)** Central slice from *C. reinhardtii* tomogram (Kelley et al., 2024, *Preprint*) (EMPIAR-11830, 573.mrc, 7.84 Å/pixel at bin4), showing the thylakoid, inner chloroplast membranes (Chl IMs), and Chl OMs. Scale bar, 100 nm. **(B)** Thickness-mapped segmentation of the chloroplast, where color values indicate mean membrane thickness. **(C)** Comparative thickness distributions for Chl OM, Chl IM, and the thylakoid. Box plots show median (center line), interquartile range (box), and whiskers extending to 1.5× the interquartile range. **(D–F)** Analysis of the Golgi apparatus membranes. **(D)** Central slice from *C. reinhardtii* tomogram (Kelley et al., 2024, *Preprint*) (EMPIAR-11830, 1570.mrc, 7.84 Å/pixel at bin4), showing the ER and Golgi cisternae from the CGN through the medial Golgi stack to TGN. Scale bar, 100 nm. **(E)** Thickness-mapped segmentation of the membranes, where color values indicate mean membrane thickness. **(F)** Thickness distribution analysis across the Golgi stack highlights distinct cisternae populations within the Golgi apparatus, with membrane thickness progressing from ER-like values in the CGN, decreasing in the medial cisternae, and then increasing toward the TGN. Box plots show median (center line), interquartile range (box), and whiskers extending to 1.5× the interquartile range.

Examining a representative *Chlamydomonas* chloroplast tomogram provided another example of thickness variations between functionally distinct membranes. We observed that the outer envelope membrane (mean thickness ≈5.9 nm) was consistently thicker than both the inner envelope and thylakoid membranes (mean thicknesses ≈4.7 nm, Fig. 7, A–C), in line with known differences in lipid composition (see Discussion).

The Golgi apparatus represents another striking example of intrinsic variations within a single organelle. Composed of a series of flattened membrane-enclosed cisternae and vesicles, it functions as the primary sorting center for protein and lipid trafficking, directing cargo from the ER to the PM and endolysosomal system. Our analysis of the *Chlamydomonas* Golgi revealed a membrane thickness gradient across the stack (Fig. 7, D–F). The membranes of the CGN exhibited low luminal protein density, similar to ER membranes, and had comparable mean thickness values (≈5.9 nm). Progressing through the flattened medial Golgi stack, luminal protein density increased, and several consecutive cisternae membranes displayed a consistently reduced mean thickness of ≈5 nm. This pattern aligns with observations that specific glycosylation enzymes distribute across two to three cisternae in specific sub-Golgi regions, depending on their position in the glycosylation pathway (Welch and Munro, 2019). In the TGN, where protein sorting for PM delivery occurs, the mean membrane thickness increased to ≈5.4 nm, coupled with a decrease in luminal protein density. These results provide independent experimental evidence supporting functional specialization across different regions of the Golgi, in line with the membrane thickness sorting hypothesis (Bretscher and Munro, 1993; Munro, 1995; Munro, 1998; Welch and Munro, 2019) (see Discussion).

## Discussion

In this study, we present a semiautomated computationally efficient workflow for measuring local membrane thickness from cryo-electron tomogram segmentations, implemented in publicly available Python code that can be integrated into existing cryo-ET analysis pipelines. Our analysis of *Chlamydomonas* and human cell tomograms reveals systematic thickness relationships across organelle membranes. Specifically, we observe that the ER and NE show similar thickness profiles, consistent with their continuous nature and similar lipid compositions (van Meer et al., 2008). In contrast, mitochondrial and thylakoid membranes are consistently thinner than the ER, Golgi, and NE, possibly due to their specialized compositions and functional roles (Fig. 5 A and Fig. 6 A). These findings align with experimental reports on organelle-specific lipid compositions and provide a first quantitative analysis of membrane thickness heterogeneity in cellular contexts.

The workflow we propose differs from previous approaches in several key aspects: Instead of confining the analysis to 2D projections, we measure membrane thickness in 3D by computing Euclidean distances between paired segmentation surface points, guided by their normal vectors, thus accounting for local membrane curvature variations. The thickness measurement step is parallelized, enabling high-throughput analysis of hundreds of thousands to millions of point-to-point distance calculations per membrane within a tomogram. Additionally, we extract intensity profiles by sampling the input tomogram along vectors between paired points in 3D and use characteristic membrane profile features as quality criteria for excluding unreliable measurements in an automated manner. The high sampling density enhances the statistics of the resulting measurements and ensures their sensitivity to both global thickness differences across organelles and local variations within individual membranes. The consistent thickness distributions for a given organelle across different orientations and tomogram acquisition conditions demonstrate the method’s robustness (Fig. 4 and Fig. S3). Finally, the resulting membrane thickness maps can be directly overlaid with the input tomogram or with coordinates of complexes identified by subtomogram averaging or template matching, enabling contextual analysis of local membrane thickness variations in relation to protein localization.

Our in silico approach using MD simulation files converted to resolution-limited EM density maps suggests that membrane proteins minimally distort the characteristic features of membrane density profiles at standard cryo-ET resolution scales (Fig. 3 and Fig. S2). Since cellular membranes are inherently composite materials of both lipids and proteins, in the case of experimental intensity profiles, we consider thickness measurements as valid representations of this composite structure as long as the presence of proteins does not hinder the identification of the features required for quality control.

The in silico analysis additionally provided insights into identifying membrane interface boundaries. Conventional approaches measure membrane thickness as the distance between intensity profile minima (Cornell et al., 2020; Heberle et al., 2020). However, projecting phosphate coordinates onto the profiles (unsurprisingly) showed that they extend onto the profile slopes, i.e., the hydrophilic layer does not sharply end at the minima. Measuring membrane thickness as the distance between the profile inflection points may encompass more of the hydrophilic region and thus better capture the membrane interface boundaries. These inflection points mark the steepest changes in electron density and broadly coincide with the segmentation boundaries from the experimental tomograms that we used for our thickness measurements (Fig. S1, A and C), suggesting that the segmentation U-Net likely identified membrane edges based on sharp contrast changes from the uneven electrostatic potential distribution across the lipid bilayer (Förster and Briegel, 2024). Similarly, Wietrzynski et al. (2025) defined membrane boundaries at the centers of profile slopes, arguing that in cryo-ET densities change gradually rather than having sharp edges. Since defining the membrane boundary remains somewhat subjective, we provide users with the option to report profile minima-to-minima distances as an alternative readout for membrane thickness.

Our in situ membrane thickness measurements are broadly consistent with previously reported bilayer thicknesses of compositionally less complex in vitro systems. Depending on the acyl chain length, the hydrophobic core thickness has been shown to vary between ≈2.5 and 4 nm, as measured by SAXS (Lewis and Engelman, 1983) and cryo-EM (Cornell et al., 2020; Heberle et al., 2020; Schönnenbeck et al., 2025), while SANS measurements have reported thickness values for the total bilayer, including the hydration layer, between ≈4.7 and 6.4 nm (Kucerka et al., 2009; Woodka et al., 2012). The cellular membrane thicknesses we obtain fall within this latter range, strengthening the notion that the input membrane segmentations capture both the hydrophobic core and hydrophilic headgroups. If the hydration layer thickness (≈1.5 nm, as determined by SANS [Kucerka et al., 2009]) is added to SAXS- and cryo-EM–derived measurements of the hydrophobic core, the resulting values closely match the thickness distributions we observe. The agreement with established experimental methods suggests that our approach can be used to confidently measure membrane thickness in native cellular environments. More recently, Medina et al. (2025, *Preprint*) also reported a cryo-ET analysis pipeline to measure membrane thickness from cellular data. This study found thickness variations across organelles, which are in qualitative agreement with our observations.

Beyond organelle-wide differences, we detect systematic thickness variations within the membranes of individual organelles. The IMM is consistently thicker than the OMM within the same tomogram (Fig. 2 E, Fig. 5 A, and Fig. 6 A), consistent with findings by Medina et al. (2025, *Preprint*). The properties of the IMM are thought to largely derive from its high cardiolipin content (≈20% of the lipid mass)—a diphosphatidylglycerol lipid with a small hydrophilic headgroup and large hydrophobic tail region that facilitates the high membrane curvature in cristae (Gaspard and McMaster, 2015). Additionally, the IMM is highly rich in protein, with respiratory complexes constituting ≈60–70% of its mass (Becker et al., 2009). Similar membrane specialization exists in chloroplasts, which are photosynthetic organelles enveloped by two membranes, whose distinct functions are dictated by the differences in lipid and protein compositions. Within the stromal volume, the protein complexes of photosystems I and II sit on the thylakoid membranes, whose lipid composition is similar to that of the inner envelope membrane (Block et al., 1983). We observe that the outer envelope membrane is thicker than both the inner envelope and thylakoid membranes (Fig. 7, A–C), which exhibit overlapping thickness distributions consistent with their similar lipid profiles (Block et al., 1983).

Perhaps most striking are the thickness gradients we observe across the Golgi. According to the membrane thickness sorting hypothesis proposed by Munro and colleagues, membrane protein localization throughout the secretory pathway primarily depends on the match between the length of the transmembrane span and the thickness of the bilayer (Bretscher and Munro, 1993; Munro, 1995; Munro, 1998; Welch and Munro, 2019). This model posits that lipid composition and membrane thickness change accordingly: CGN cisternae should be surrounded by thin, phospholipid-rich membranes with low cholesterol content, similar to the ER, while TGN cisternae membranes should become progressively thicker as sphingolipid and sterol content increases (Holthuis et al., 2001). Sphingolipid synthesis in the Golgi drives cholesterol enrichment through non-vesicular transport from the ER (Holthuis et al., 2001). This increasing membrane thickness from CGN to TGN would favor trafficking of proteins with longer transmembrane spans to the PM, as supported by immunofluorescent experiments showing that PM proteins with synthetic transmembrane domains of 23 leucines reach the cell surface, while those with truncated domains (17 leucines) accumulate in the Golgi (Munro, 1995).

Our data provide orthogonal evidence for key aspects of the membrane thickness sorting mechanism. We observe that membrane thickness increases in discrete steps from medial Golgi toward TGN: medial cisternae exhibit higher luminal density (likely representing Golgi-resident proteins) and thinner membranes compared with TGN cisternae, which have lower luminal protein density and thicker membranes (Fig. 7, D–F). These findings are consistent with work by Bykov et al. (2017), who documented similar changes in luminal density and relative membrane thickness across the Golgi. We observed that the ER and CGN displayed similar mean thicknesses, although both were thicker than the medial Golgi. The pattern of increasing thickness from medial to TGN supports the thickness-based protein sorting principle. How these thickness relationships relate to the functional mechanism of protein sorting in the Golgi remains to be investigated.

Despite these promising results, our method has the following limitations: First, the input tomograms require a sufficient signal-to-noise ratio for optimal segmentation results, and manual curation of instance segmentation labels may occasionally be necessary. Second, thickness estimation becomes less reliable in regions with extreme curvature changes (e.g., on self-folding membranes) due to reduced surface point coverage and potential inaccuracies in normal vector assignments. However, the automated results filtering step and the high number of individual measurements across each membrane typically mitigate these local inaccuracies, maintaining statistical robustness. Third, the approach is constrained by the voxel size of input tomograms; therefore, thickness variations should be interpreted across sufficiently large membrane patches rather than at individual point pairs to avoid misinterpretation. Fourth, membrane thickness serves only as an indirect proxy for compositional differences across organelle membranes. While membrane intensity profiles are dominated by lipid phosphate headgroups, membrane proteins contribute electron optical density that might subtly alter profile features (e.g., peak width and minima positions), which could in turn influence the segmentation boundaries and thickness measurements. Thickness measurement values therefore likely reflect the combined contribution of lipids and proteins, though lipids remain the primary determinant. A more comprehensive understanding of membrane organization can be achieved by using our method as a complementary approach to other techniques, such as high-resolution correlative light and EM workflows with chemically modified lipid probes (Lennartz et al., 2025, *Preprint*).

At a finer scale, lipid composition is thought to vary across the leaflets of a single membrane, a phenomenon known as lipid asymmetry, and laterally within membranes, referred to as microdomains (Devaux and Morris, 2004). While the ER maintains relative compositional equilibrium between its leaflets through ATP-independent transporters, the Golgi, PM, and endosomal membranes exhibit compositional asymmetry (Devaux and Morris, 2004). To probe for such asymmetry effects, we used the experimentally extracted intensity profiles and compared the relative depths of each minimum with the central maximum for the two PM leaflets, following a similar approach by Heberle and Doktorova (2025).

Our cholesterol depletion experiment provided an opportunity to probe for changes related to both membrane thickness and asymmetry. Independent of tomogram preprocessing, we observed trends toward increased membrane thickness and reduced PM leaflet asymmetry under cholesterol depletion conditions. Even though our limited number of PM instances (four per condition) prevents definitive conclusions (Fig. 6, C–F and Fig. S4), the trend toward reduced leaflet asymmetry aligns with reports on cholesterol’s central role in maintaining asymmetric lipid distributions across cellular PM leaflets (Doktorova et al., 2025). Although the thickness increase may appear unexpected compared with prior in vitro results (Kucerka et al., 2009), we note that cholesterol-depleted cells displayed morphological changes, likely indicating a complex cellular response to the acute treatment. Previous research has shown that cholesterol depletion impedes lateral diffusion of transmembrane proteins due to cortical actin cytoskeleton reorganization (Kwik et al., 2003), thus our perturbation experiment likely affected multiple cellular systems rather than lipid composition alone. Alternatively, the observed thickening of the PM could reflect aberrant clustering of membrane proteins into patches on the cell surface, similarly to Hao et al. (2001). While these preliminary findings demonstrate that our workflow can detect both thickness and asymmetry changes in perturbation experiments, future studies will be required to draw definitive conclusions and disentangle complex cellular responses from direct effects of lipid composition changes.

Our workflow for measuring membrane thickness from cryo-electron tomogram segmentations offers a systematic framework to quantify membrane thickness variations in native cellular contexts. It provides an orthogonal tool to address open questions in membrane biology. For example, building on our findings on the thickness gradients across the Golgi membranes, a similar approach can be applied to examine how disruptions in the protein sorting machinery affect membrane architecture in ER or Golgi trafficking mutants. Combined with perturbation experiments, our workflow can provide a direct readout of the effects of temperature changes, pharmacological treatments, or genetic modifications of lipid metabolism on membrane organization and thickness. Furthermore, integrating measurements of thickness variations within membrane microdomains with co-localization data for specific lipid and protein species can offer an unbiased in situ approach to testing the lipid raft hypothesis. Finally, a community-driven effort could enable broader taxonomic sampling to assess whether the patterns observed in *Chlamydomonas* and human cells are conserved across different organisms. With its publicly available code and accompanying tutorial, we anticipate that our approach has the potential to become a valuable tool for the community, enabling researchers to probe questions related to membrane organization in situ.

## Materials and methods

### Cell culture

Human embryonic kidney HEK293 (HEK Flp-In T-Rex 293, Invitrogen) cells were cultured in DMEM (Sigma-Aldrich) supplemented with 10% FBS (Gibco) under standard tissue culture conditions (37°C, 5% CO_2_) in T75 or T25 cell culture flasks (Greiner Bio-One).

### Pharmacological cholesterol depletion

Acute cholesterol depletion was induced in HEK293 cells using 10 mM MBCD (Sigma-Aldrich). Cells were seeded on EM grids or in 6-well plates. For cholesterol depletion, cells were incubated for 30 min in FBS-free DMEM containing 10 mM MBCD and 25 mM HEPES buffer. Control cells were treated with FBS-free DMEM supplemented with 25 mM HEPES alone for 30 min. After the treatment, cells were either plunge-frozen for further cryo-EM sample preparation or washed and pelleted for cholesterol quantification.

### Cholesterol quantification

Cholesterol levels in MBCD-treated and control HEK293 cells were measured using a fluorometric assay kit (Amplex Red; Thermo Fisher Scientific) in duplicate for each condition. HEK293 cells were seeded in 6-well plates overnight, treated as described, washed with PBS, trypsinized, and resuspended in DMEM. Cell suspensions were used to obtain cell counts using an automated counter (CellDrop, DeNovix). Cells were then gently pelleted in a pre-cooled centrifuge at 4°C, and the cell culture media was removed. Cell pellets were lysed in detergent-containing buffer (kit-supplied) by vortexing, two freeze–thaw cycles, and gentle sonication. Soluble protein concentration was measured using the bicinchoninic acid (BCA) assay (Pierce BCA Protein Assay Kit; Thermo Fisher Scientific). Fluorescence in the lysed samples was generated by initiating enzyme-coupled reactions following cholesterol oxidation (all following the manufacturer’s instructions) and measured in light-impermeable 96-well plates with an automated plate reader (Tecan). Cholesterol concentrations in the cell samples were determined by normalizing fluorescence values to a standard curve. Cholesterol levels were normalized to either soluble protein content or cell count (Fig. S4 A).

### Cryo-EM sample preparation

EM support grids (Au, 200 mesh, R2/2, SiO_2_ foil; Quantifoil) were glow discharged using a Pelco easiGlow Discharger (15 mA, 90 s). The foil side of the grids was functionalized by incubation with 30 µg/ml laminin (catalog number 11243217001; Roche) for 1 h at room temperature or overnight at 4°C. Excess laminin solution was aspirated, the grids were washed twice with PBS and placed in a 35-mm cell culture dish containing DMEM and FBS. Adherent HEK cells (3–5 × 10^4^ cells/ml) were seeded on the foil side of grids and allowed to attach overnight. Cells were treated as described, back-blotted to remove excess media, and vitrified by plunge freezing into liquid ethane with a Leica EM GP2 plunger. The grids were assembled into AutoGrid cartridges (Thermo Fisher Scientific). To obtain electron-transparent sections of the sample, lamellae were prepared by cryo-focused ion beam (FIB) milling on a dual-beam Aquilos FIB-scanning electron microscope (SEM) (Thermo Fisher Scientific), equipped with a gallium ion source, similarly to a previously described protocol (Klumpe et al., 2021). Briefly, samples were coated with an organometallic platinum layer via a gas injection system and sputter coated with platinum (20 s, 1 kV, 10 mA). Automated rough milling was performed using the AutoTEM 5 Software (Thermo Fisher Scientific), with stepwise reductions in the milling current and the distance between milling patterns. Fine milling was performed manually at 10–30 pA with milling patterns spaced 130–200 nm apart. The milling process was guided by SEM imaging (13 pA 2–10 kV). A final platinum sputter coat (2 s,1 kV, 10 mA) was applied at the end of the session.

### Cryo-electron tomogram acquisition

The tilt series used for membrane thickness analysis in control and cholesterol-depleted HEK293 cells were acquired from three grids across three acquisition sessions (two for control and one for cholesterol-depleted cells). Data were collected on a Titan Krios G4 transmission electron microscope, equipped with a cold field emission gun and Falcon 4i direct electron detector, operated at 300 kV acceleration voltage in counting mode (Thermo Fisher Scientific). Suitable acquisition areas on each lamella were selected with overview images with 3.0 nm pixel size. Tilt series were collected using a dose-symmetric scheme (Hagen et al., 2017) in 2° increments, grouped by two, over a ±60° range with a target cumulative electron dose of 120–130 e^−^/Å^2^. Projection images were recorded at 64k magnification, corresponding to a pixel size of 1.97 Å, with a 10-eV wide energy slit inserted and nominal defocus varied in 0.5 µm steps in the range of −2.5 to −4.5 µm. Each projection image was acquired in low-dose mode as a 4,096 × 4,096 pixel 10-frame movie, with frames motion corrected and aligned on the fly in SerialEM (Mastronarde, 2005).

### Tomogram reconstruction

Tilt series were preprocessed using the cryoKIT_10_ toolkit that combines multiple common tools used for cryo-ET processing. Individual tilt images were visually assessed, and those with substantial ice reflections, drifts, or lamella edge obstructions were excluded. Additionally, tilt images with defocus values (estimated with Gctf v1.06 [Zhang, 2016]) that deviated significantly from the mean were discarded, regardless of perceived visual quality. The remaining tilt images were dose filtered based on cumulative electron dose (Grant and Grigorieff, 2015), as previously described (Wan et al., 2017). Cleaned, dose-filtered tilt series were aligned using patch tracking in AreTomo2 (Zheng et al., 2022). Tomograms were reconstructed at bin4 using the WBP method (Radermacher, 1992) implemented in AreTomo2.

### Contrast enhancement via denoising and deconvolution filtering

To enhance contrast and improve segmentation accuracy of HEK293 cell tomograms, denoising was performed using the cryoCARE U-Net (Buchholz et al., 2019), applied to even and odd movie frames. Tomograms were then reconstructed using the WBP method implemented in AreTomo2, as described. cryoCARE-denoised *C. reinhardtii* tomograms were obtained from EMPIAR-11830 (Kelley et al., 2024, *Preprint*).

### Instance membrane segmentation

Membrane segmentations were generated using MemBrain-seg U-Net with the pre-trained MemBrain_seg_v10_alpha.ckpt model, applying the connected components analysis option to generate unique labels for individual membrane instances within the tomographic volume (Lamm et al., 2025, *Preprint*).

In certain cases, segmentation labels were manually curated in napari (Chiu et al., 2022) by using the seg-select plugin (Wimmer, 2025) to merge segmentation labels (e.g., to combine individual cristae in a single label for IMM) or using the Lasso tool in the updated MemBrain plugin (Lamm et al., 2025, *Preprint*) to isolate membrane instances (e.g., to split the inner and outer NE membranes). The instance segmentation labels were assigned to their respective membrane instances based on human judgment.

For visualization, segmentations were rendered in ChimeraX (Pettersen et al., 2021) and color-coded either according to their membrane identity (Figs. 1, 2, 4, and S3) or to their mean membrane thickness (Fig. 7).

### Membrane thickness analysis

The procedure for membrane thickness measurements was described in detail in the main text in section “A computational workflow for in situ membrane thickness analysis” and is visualized in Fig. 1. The complete Python source code (memthick.py) and a detailed tutorial are available in the Contextual Analysis Tool for cryo-ET (cryoCAT) GitHub public repository (Turoňová et al., 2025). The tutorial guides users through the workflow options and key parameters and demonstrates how to reproduce the manuscript figures, including 3D visualizations, using their own data.

Representative processing times for membrane segmentations from bin4 tomograms are shown in Fig. S3 F. Prior to running the pipeline, manual curation of segmentation labels (e.g., merging or splitting instances) typically required 5–30 min per segmentation volume depending on the number and geometric complexity of the membranes of interest. As for the running the thickness pipeline itself, performance benchmarks were conducted on two systems: GPU-accelerated processing was done on a Linux system with x86 CPUs and an NVIDIA A100 GPU, while CPU-only processing was performed locally on a macOS system with ARM-based M-series processors using four cores. Only the point-pair thickness measurement step is parallelized and benefits from the GPU acceleration; surface point reconstruction and intensity profile filtering run on single CPU cores and show similar performance regardless of the processing system.

### Statistical analysis

For analyzing the mean thickness values plotted in Fig. 5; and Fig. 6, A and B, we employed two-sided paired or unpaired *t* tests. Membrane thickness was calculated from paired point-to-point measurements. For each membrane instance, we first calculated the mean thickness value from thousands to millions of individual point pair measurements. Each instance was treated as an independent biological replicate. For functionally related membranes acquired within the same tomographic volume (OMM vs IMM, thylakoid vs Chl OM, ER vs CGN, ER vs medial Golgi, CGN vs medial Golgi, and medial Golgi vs TGN), we used two-sided paired *t* tests comparing these instance-level mean values. For membrane types observed in different tomograms (NE vs ER), we applied two-sided unpaired *t* tests using the same instance-level means. The resulting P values were interpreted using standard significance thresholds (ns: P > 0.05; *: P < 0.05; **: P < 0.01; ***: P < 0.001). The data points in Fig. 5 A represent mean thickness values for each membrane instance from the *Chlamydomonas* dataset: NE (*n* = 10 instances, 2.2 M point pair measurements), ER (*n* = 20 instances, 3.1 M point pair measurements), OMM (*n* = 19 instances, 2.5 M point pair measurements), IMM (*n* = 19 instances, 6.5 M point pair measurements), thylakoid (*n* = 15 instances, 21.4 M point pair measurements), and Chl OM (*n* = 9 instances, 1.2 M point pair measurements). For the Golgi apparatus (*n* = 4 tomograms, 35 cisternae, 4.3 M point pair measurements), mean thicknesses were calculated for each individual cisterna. Cisternae were manually assigned to either CGN, medial Golgi, or TGN. We applied two-sided paired t-test for statistical comparison between the different Golgi compartments. Similarly, the data points in Fig. 6 A represent mean thickness values for individual membrane instances in HEK293 tomograms: NE (*n* = 4 instances, 0.7 M point pair measurements), ER (*n* = 6 instances, 0.4 M point pair measurements), PM (*n* = 4 instances, 0.5 M point pair measurements), OMM (*n* = 9 instances, 0.8 M matched measurements), and IMM (*n* = 9 instances, 3.2 M point pair measurements). All statistical analyses were performed using the scipy.stats package (Virtanen et al., 2020). Data distribution was assumed to be normal, but this was not formally tested.

### Converting MD simulation boxes to EM volumes

To investigate the effects of membrane proteins on EM density profiles and downstream membrane thickness measurements for proteins that fall below the resolution limit of individual experimental cryo-electron tomograms, we converted published publicly available MD simulation files (Goretzki et al., 2023; Schaefer and Hummer, 2022) to EM density maps using a custom Python script based on the EMAN2 e2pdb2mrc.py framework (Tang et al., 2007).

#### Density map generation

Atomic coordinates from .gro simulation files were converted to EM density maps using electron scattering factors for amplitude-based contrast and weak phase object approximation combining amplitude and phase information. For the weak phase method, each atom contributed a complex value *A*exp(*iφ*), where amplitude *A* derives from electron scattering factors (values approximated based on Mott scattering cross sections) and phase *φ* from (approximate) electrostatic potentials scaled by a phase strength parameter (0.2). The imaginary component was extracted to simulate phase contrast conditions typical for EM (Förster and Briegel, 2024). Density maps were converted to NumPy arrays for subsequent analysis (Harris et al., 2020).

#### Spatial resolution

To simulate finite resolution effects of EM, each atom was represented as a 3D Gaussian blob rather than a point scatterer, similarly to Tang et al. (2007). Gaussian width was set to achieve a target resolution of 1.6 Å, corresponding to relatively high-magnification acquisition for cryo-ET data, with *σ* = FWHM/2.35.

#### Protein localization and spatial binning

The center of mass of all protein atoms was calculated in the XY plane to establish the reference point for distance analyses of the effect of the protein on the density plot (illustrated in Fig. 3 C and Fig. S2 C). Concentric annular rings were defined around the protein center, creating distance bins (typically 0–1 nm, 1–2 nm, 2–3 nm, etc.) from which corresponding membrane density profiles were extracted.

#### Density profile extraction

Within each distance bin, membrane density profiles were extracted perpendicular to the membrane plane by averaging the density map over all positions within that annular ring. The resulting density profiles exhibited the characteristic membrane signature: two minima marking the phosphate-rich headgroup regions separated by a maximum corresponding to the hydrophobic core.

#### Membrane thickness measurement approaches

Thickness was quantified using the (1) minima-to-minima distance (thickness is defined as the distance between the two headgroup minima in each profile) and (2) the distance between inflection points on the profile slopes (thickness is measured between the first density gradient extrema found outward from each headgroup minimum). The density gradient captures the rate of change of density with position: while the gradient equals zero at density maxima and minima (where the rate of change is zero), the gradient extrema themselves mark locations of steepest density change—typically at interface boundaries where density undergoes sharp transitions. Beginning at each headgroup minimum position (where density gradient = 0), the analysis protocol identifies the immediately adjacent gradient extremum on each side—the first minimum or maximum encountered when moving outward from each peak headgroup region.

#### Phosphate position analysis

Phosphate coordinates were extracted using the MDAnalysis Python package (Gowers et al., 2016; Michaud-Agrawal et al., 2011). Phosphate positions were projected onto the in silico density profiles to analyze their spatial distribution relative to the positions of both density minima and the adjacent inflection points (yellow points in Fig. 3 D and Fig. S2 D).

### Membrane orientation relative to the missing wedge

To assess whether there is orientation-dependent bias in the thickness measurements, we calculated the angle between membrane normal vectors and the missing wedge direction for each measurement point pair. The missing wedge direction was defined as the z-axis in tomogram coordinates. The normal vector of each measurement point was extracted from the outputted .csv measurement files, and its orientation angle with respect to the missing wedge, θ, was calculated as θ = arccos(|n · z|), where n is the normalized membrane normal vector and z is the unit vector along the missing wedge axis. This yields angles from 0° (membrane parallel to missing wedge, most affected by missing information) to 90° (membrane perpendicular to missing wedge, least affected). The absolute value ensures that angles represent the acute angle regardless of normal vector direction. To identify systematic bias introduced by the missing wedge artifact, we computed Pearson correlation coefficients between orientation angles (θ) and corresponding thickness measurements for each membrane dataset using NumPy (Harris et al., 2020). Correlation coefficients near zero would indicate the absence of orientation-dependent bias, while significant positive or negative correlations would suggest systematic effects of the missing wedge on thickness measurements.

### Asymmetry analysis of the intensity profile peaks

To quantify asymmetry in experimentally derived intensity profiles, we compared the relative depths of each headgroup minimum to the central hydrophobic core maximum, following a similar approach by Heberle and Doktorova (2025). The thickness measurement pipeline generates .pkl files containing both the spatial positions (in tomogram coordinates and projected onto the extracted profile) and intensity values of the two headgroup minima and central hydrophobic maximum for each measurement point pair. To minimize noise from individual measurements, we implemented a thickness-based binning approach. Profiles were grouped into bins (typically 0.1 nm wide) based on the membrane thickness measured between the paired points. Only bins containing a minimum number of profiles (typically ≥20) were included in the subsequent analysis. Within each thickness bin, profile features were aggregated to reduce the effect of outliers. An asymmetry score was calculated as the ratio of intensity depth differences between the central maximum and each headgroup minimum: (|central_max_intensity - minima1_intensity| vs |central_max_intensity - minima2_intensity|). The score was computed by dividing the larger by the smaller difference to ensure scores ≥1.0. A score of 1.0 indicates perfect symmetry between the two leaflet intensities, while scores >1.0 reflect increasing asymmetry. Results in Fig. 6 F; and Fig. S4, H and I are presented as percentage asymmetry using (asymmetry_score - 1.0) × 100% to provide a more intuitive interpretation, where 0% indicates perfect symmetry and higher percentages reflect greater leaflet asymmetry.

### Online supplemental material

Fig. S1 shows IMM thickness values pre- and post automated filtering based on identifying features from the tomogram intensity profiles, including a spatial distribution of the results. Fig. S2 shows thickness analysis of MD simulated membrane density profiles in the vicinity of TRPV4 channel. Fig. S3 shows orientation-dependent analysis of NE membrane thickness measurements across four tomograms, which finds no correlation between membrane orientation relative to the missing wedge and measured thickness. Fig. S4 shows comparison of PM thickness and asymmetry distributions in control versus cholesterol-depleted HEK293 cells using different tomogram reconstruction methods. Video 1 shows detailed visualization of the local membrane thickness map in Fig. 2 C with overlaid ATP synthase coordinates on the IMMs.

## Data Availability

Representative cryoCARE-denoised bin4 tomograms of HEK293 cells containing PMs in the field of view have been deposited at the EM Data Bank for control (EMD-55308) and cholesterol-depleted (EMD-55310) cells. Tilt series (raw and dose-filtered), acquisition metadata files, tilt angle files, CTF estimation outputs, AreTomo2 alignment files, and IMOD transform files are available at the EMPIAR under accession code EMPIAR-13031. Source data for all figures (.csv files with point pair positions, orientations, distance measurements, and .pkl files for intensity profiles), organized by figure number, are available through the Zenodo repository https://doi.org/10.5281/zenodo.17286398. The published tilt series and cryoCARE-denoised bin4 tomograms for *C. reinhardtii* are available under accession code EMPIAR-11830. The consensus subtomogram average map of the *Chlamydomonas* ATP synthase is deposited under the accession code EMD-51802. The annotated ATP synthase positions and orientations are available through the public repository: https://github.com/Chromatin-Structure-Rhythms-Lab/ChlamyAnnotations/tree/master/10.1101-2024.12.28.630444 (“star” folder) (Righetto et al., 2025). The MD simulation data from studies (Schaefer and Hummer, 2022; Goretzki et al., 2023) are available through the following Zenodo repositories: https://doi.org/10.5281/zenodo.6797842 and https://doi.org/10.5281/zenodo.7957940, respectively. The source code used in this study is part of the cryoCAT public GitHub repository (Turoňová et al., 2025), specifically available at https://github.com/turonova/cryoCAT/blob/main/cryocat/memthick.py. A Jupyter notebook, outlining the usage of the code along with detailed documentation, can be found at https://github.com/turonova/cryoCAT/blob/main/docs/source/tutorials/membrane_thickness/measure_thickness_tutorial.ipynb.
